# Pyrin-only protein 2 limits inflammation but improves protection against bacteria

**DOI:** 10.1038/ncomms15564

**Published:** 2017-06-05

**Authors:** Sivakumar Periasamy, Kristen A. Porter, Maninjay K. Atianand, Hongnga T. Le, Sarah Earley, Ellen B. Duffy, Matthew C. Haller, Heather Chin, Jonathan A. Harton

**Affiliations:** 1 Department of Immunology and Microbial Disease, Albany Medical College, Albany, New York 12208 USA; 2 Department of Biochemistry, University of Science, Vietnam National University, Ward 4, District 5, Ho Chi Minh City 749000, Vietnam

## Abstract

Pyrin domain-only proteins (POPs) are recently evolved, primate-specific proteins demonstrated *in vitro* as negative regulators of inflammatory responses. However, their *in vivo* function is not understood. Of the four known POPs, only POP2 is reported to regulate NF-κB-dependent transcription and multiple inflammasomes. Here we use a transgenic mouse-expressing *POP2* controlled by its endogenous human promotor to study the immunological functions of POP2. Despite having significantly reduced inflammatory cytokine responses to LPS and bacterial infection, POP2 transgenic mice are more resistant to bacterial infection than wild-type mice. In a pulmonary tularaemia model, POP2 enhances IFN-γ production, modulates neutrophil numbers, improves macrophage functions, increases bacterial control and diminishes lung pathology. Thus, unlike other POPs thought to diminish innate protection, POP2 reduces detrimental inflammation while preserving and enhancing protective immunity. Our findings suggest that POP2 acts as a high-order regulator balancing cellular function and inflammation with broad implications for inflammation-associated diseases and therapeutic intervention.

## Abstract (web summary)

Pyrin-only proteins (POPs) are primate-specific negative regulators of inflammasome activation. Here the authors generate transgenic mice expressing POP2 under the control of the human promoter, and show that POP2 is important for balancing antibacterial inflammatory responses *in vivo*.

Inflammation is critical for clearing infections and responding to injury. However, excessive or prolonged inflammation contributes to irreversible tissue pathology and organ dysfunction. Cellular and physiologic aspects of inflammation are mediated by various pro-inflammatory cytokines in response to signals provided by cell surface, endosomal or cytoplasmic pattern recognition receptors, including Toll-like (TLR) and NOD-like receptors (NLR)^1^. Most pro-inflammatory cytokines require activation of the NF-κB and/or MAPK signalling pathways and are active on synthesis, while release of IL-1β and IL-18 requires enzymatic maturation by caspase-1 via activation of an inflammasome^2^,^3^,^4^. Inflammasome assembly is initiated by cytosolic sensors belonging to the NLR or PYHIN family of proteins^4^. Although NLRs (for example, NLRP3 and NLRC4) require an evolutionarily conserved Pyrin domain (PYD) or caspase recruitment domain (CARD), PYHIN family members (for example, AIM2) rely solely on a PYD^3^. Homotypic PYD–PYD interaction between the sensor and apoptotic speck-like protein containing a CARD (ASC) is followed by recruitment and cleavage of pro-caspase-1 via a CARD–CARD interaction^2^. Multi-step processing of IL-1β and IL-18 illustrates the potential for overproduction driving harmful effects and highlights the utility of regulatory molecules to further constrain activation and release.

Viral and mammalian Pyrin domain-containing proteins (PYDC) or Pyrin-only proteins (POPs), comprised of essentially a solitary PYD, have been identified as regulators of inflammatory processes by either inhibiting NF-κB p65 signalling, limiting inflammasome formation or both^5^,^6^,^7^,^8^,^9^,^10^,^11^,^12^,^13^,^14^. Among mammalian species, POPs are evolutionarily recent, highly conserved and restricted to higher primates, implying a unique function for these proteins in modulating inflammation^10^. Four POP protein family genes (*PYDC1, PYDC2, POP3* and *NLRP2B*) and their proteins have been characterized *in vitro*^5^,^7^,^12^,^13^,^14^. POP1 (PYDC1) and POP3 (W6CW81) have highly specific functions including regulating the NLRP3 and Aim2 inflammasome, respectively^12^,^13^, while POP4 (NLRP2B) inhibits NF-κB but is unlikely to inhibit inflammasomes^13^. By contrast, POP2 (PYDC2) is thought to limit both NF-κB transactivation and NLRP3 inflammasome assembly^7^,^9^,^10^. While all POPs are expressed in macrophages, POP2 is also expressed in other haematopoietic lineages^7^.

Although transgenic mice have been developed that constitutively express POP1 and POP3 in myeloid cells; however, there is no mouse model for POP2 function, nor a model of POP function where POP inducibility and tissue specificity resemble that in humans. To explore the *in vivo* immunologic function of POP2, we developed transgenic mice-expressing *POP2* (*PYDC2*) under the control of its endogenous human promoter (POP2 mice). POP2 mice are viable, fertile and express POP2 in cells and tissues reflecting known human expression patterns. The inflammatory cytokine response to lipopolysaccharide (LPS) challenge or bacterial infection is markedly reduced in POP2 mice. Despite diminished levels of tumour-necrosis factor (TNF), IL-1β, IL-6, IL-12, IL-18 and MCP-1, and decreased neutrophil recruitment to the infection site, resistance to pulmonary tularaemia is increased. Reduced bacterial burden early during infection, elevated production of IFN-γ, improved IFN-γ-induced macrophage maturation and reduced pathology accompany this enhanced resistance, which is reversed by neutralization of IFN-γ. Thus, POP2 has a unique function in balancing detrimental and protective inflammatory responses during infection, thereby highlighting the importance of this human protein in inflammatory and infectious disease.

## Results

### Development of an endogenously regulated *POP2* transgene

In human and transfected mouse cell lines, POP2 inhibits both NF-κB/p65 transcriptional activity and NLRP3 inflammasome formation thus reducing production of inflammatory cytokines TNF and IL-1β after TLR stimulation or infection^7^,^9^. That these functions fully reflect the immunologic contribution of POP2 *in vivo* is unlikely. Further, whether similar regulation occurs *in vivo* and how POP2 influences *in vivo* inflammatory responses are unknown. Having evolved ∼25 million years ago, *POP2* is absent from the genomes of earlier mammalian species such as mice and rats^10^. The most biologically relevant transgenic mouse model of human POP2 would reproduce the human pattern of constitutive and inducible POP2 expression. Thus, we sought first to understand the genetic elements controlling POP2 expression. Upstream of the *POP2* ATG start codon, we identified two NF-κB consensus binding sequences and a putative TATA box element at −24, −36 and −82 nt, respectively (Supplementary Fig. 1A), consistent with strong induction of *POP2* mRNA in primary human monocytes and THP-1 cells on LPS, phorbol myristate acetate or TNF treatment^9^. Two putative initiator elements were found at −70 and −46 nt. In HEK293T cells, co-transfection of the NF-κB p65/RelA subunit induced a threefold increase in transcription from a luciferase reporter containing 2,000 bp of POP2 upstream genomic sequence (POP2-Luc(−2,000)) (Supplementary Fig. 1B), demonstrating that the *POP2* promoter is NF-κB responsive. Comparable luciferase activity was observed using POP2-Luc (−2,000) 5′ truncation mutants at −505, −250 or −127 (Supplementary Fig. 1C), consistent with an NF-κB consensus downstream of −127. Mutating the TATA box sequence at −82 bp reduced p65-driven luciferase activity by 2.5-fold (Supplementary Fig. 1D) suggesting TFIID-dependent initiation of *POP2* transcription, likely from a proximal, downstream transcriptional start site^15^. Although further study will be informative, the promoter elements important for *POP2* expression appear to reside within <2,000 bp immediately upstream of the POP2 coding sequence.

Treatment of THP-1 or U937 cells with the translation inhibitor cycloheximide^16^ leads to early and sustained *POP2* mRNA expression (Supplementary Fig. 1E,F). This result suggests continuous basal transcription and rapid post-transcriptional degradation of *POP2* mRNA, consistent with the rapid induction of *POP2* mRNA (∼10 min) in THP-1 and human monocytes by LPS^10^. In the presence of the RNA polymerase II inhibitor, actinomycin D (ActD)^17^, *POP2* mRNA in LPS-primed THP-1 cells declined to near baseline by 30 min (Supplementary Fig. 1G), indicating rapid turnover of *POP2* mRNA with an estimated half-life of 18 min in THP-1 cells. ActD treatment before LPS priming completely inhibited POP2 mRNA expression (Supplementary Fig. 1H), thus ActD-induced loss of RNA destabilizing proteins is not a complicating factor. Many inflammation-related genes are regulated post transcriptionally by 3′ untranslated regions (3′UTR) containing AU-rich elements (AREs)^18^,^19^. Consistent with rapid turnover and cycloheximide stabilization of *POP2* mRNA, the *POP2* 3′UTR contains a potential, TNF-like, class III ARE (AUUUUA). The *POP2* 3′UTR dramatically reduced SV40-driven luciferase expression, while mutation of the putative ARE partially restored luciferase activity (Supplementary Fig. 1I), confirming that a *cis*-acting 3′ARE partially control *POP2* mRNA stability. These results provide initial insight into post-transcriptional control of *POP2* expression and strongly suggest that appropriate *in vivo* regulation requires the *POP2* 3′UTR.

LPS induction of the HA-tagged transgene containing 2,000 bp of upstream sequence, POP2 coding sequence and downstream 3′UTR was confirmed in stable J774A.1 and RAW264.7 mouse macrophage cell line transfectants (Supplementary Fig. 2A,B). LPS-induced TNF production was diminished in POP2 transgenic J774 cells and IL-1β was dramatically reduced on treatment with LPS plus the Nlrp3 agonists adenosine tri-phosphate (ATP) or nigericin (Supplementary Fig. 2C). As the *POP2* transgene appeared sufficient to maintain regulation of POP2 expression and recapitulate its previously described functions in mouse cells, the construct was used to produce transgenic mice.

### POP2 expression is similar in humans and transgenic mice

**Figure 1:**
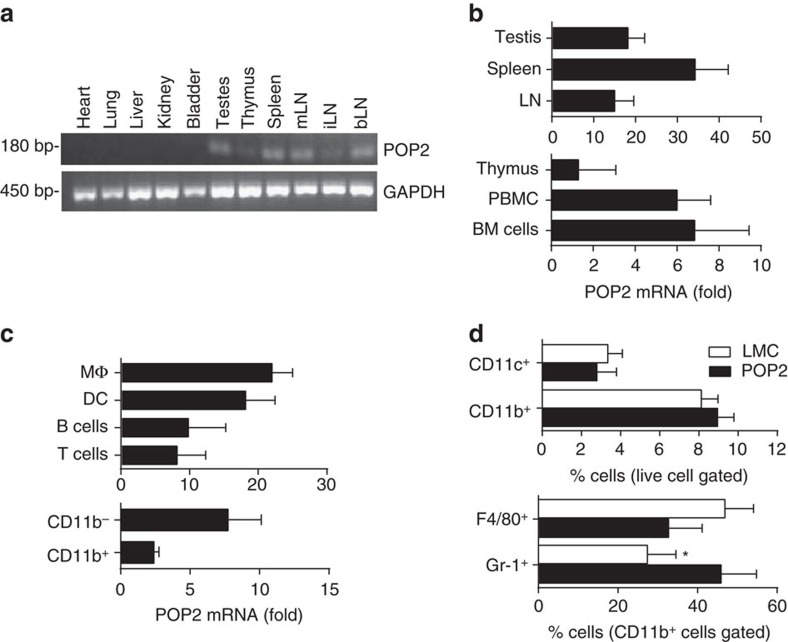
Expression of POP2 in transgenic mice. (**a**) Detection of POP2 Tg expression by RT–PCR in perfused organs (Supplementary Fig. 3). (**b**,**c**) Relative POP2 Tg basal mRNA levels (normalized to GAPDH) in various tissue and cellular compartments by qPCR. The data are shown as mean fold change relative to LMC±s.d. (*n*=4). (**d**) The frequency (%) of major myeloid-lineage cells (CD11c^+^ dendritic cells, CD11b^+^ myeloid cells, F4/80^+^ macrophages (CD11b^+^CD11c^−^Gr-1^−^F4/80^+^) and Gr-1^+^ neutrophils (CD11b^+^CD11c^−^Gr-1^+^F4/80^−^)) in spleen. The data are shown as mean±s.d. (*n*=4). To determine significance, Student’s *t*-test was performed. *P<0.05. LN, lymph nodes; mLN, mesenteric LN; iLN, inguinal LN; bLN, brachial LN; BM, bone marrow.

In humans, POP2 is expressed constitutively and abundantly in the testes, to a lesser extent in lymphocytes, but is highly inducible in myeloid cells^7^,^10^,^20^. In POP2 transgenic mice, *POP2* is expressed constitutively in the testes, spleen, lymph nodes and thymus (Fig. 1a). Subsequent real-time polymerase chain reaction (PCR) analysis confirmed abundant *POP2* mRNA in testes, spleen and lymph nodes (Fig. 1b), with lower expression in thymus, peripheral blood leukocytes and bone marrow cells. *POP2* mRNA was also highly expressed in isolated splenic macrophages, dendritic cells and to a lesser extent in B, T and CD11b^−^ and CD11b^+^ populations (Fig. 1c). POP2 protein was expressed in both myeloid and lymphoid cells from spleen and bone marrow (Supplementary Fig. 3). Further, *POP2* mRNA was induced in POP2 mouse primary macrophages after LPS treatment (Supplementary Fig. 4). Thus, the basic pattern of POP2 expression in POP2 mice reflects that of human cells and tissues.

Frequencies of CD4^+^ or CD8^+^ T cells, B cells and NK1.1 cells from primary and secondary lymphoid tissues, CD4^+^CD25^+^FoxP3^+^ Tregs and CD4^+^IL-17^+^ T cells from lymph nodes and spleen, and major myeloid-lineage cells from the bone marrow of POP2 mice were comparable to littermate controls (LMC) (Supplementary Fig. 5A). However, while overall frequencies of CD11c^+^ and CD11b^+^ cells in the spleen were comparable, CD11b^+^ Gr-1^+^ neutrophils were significantly increased (Fig. 1d and Supplementary Fig. 5B) with a corresponding, but less substantial, decrease in CD11b^+^ F4/80^+^ Gr-1^−^ macrophages. Thus, the POP2 transgene has little impact on the frequencies of most major immune cell populations in naive mice. Evaluating how POP2 influences the resting pool of splenic neutrophils will require further study.

### POP2 reduces macrophage inflammatory cytokine production

Whether, and to what extent, POP2 regulates the pro-inflammatory cytokine responses of different types of primary macrophages is unknown. Therefore, the responses of POP2 transgenic primary macrophages to LPS stimulation, Nlrp3 inflammasome activation and bacterial infection were evaluated. Bone marrow-derived (BMDM), peritoneal (PM) and isolated splenic macrophages from LMC and POP2 mice were stimulated with LPS alone or with LPS followed by the Nlrp3 inflammasome agonist ATP.

**Figure 2:**
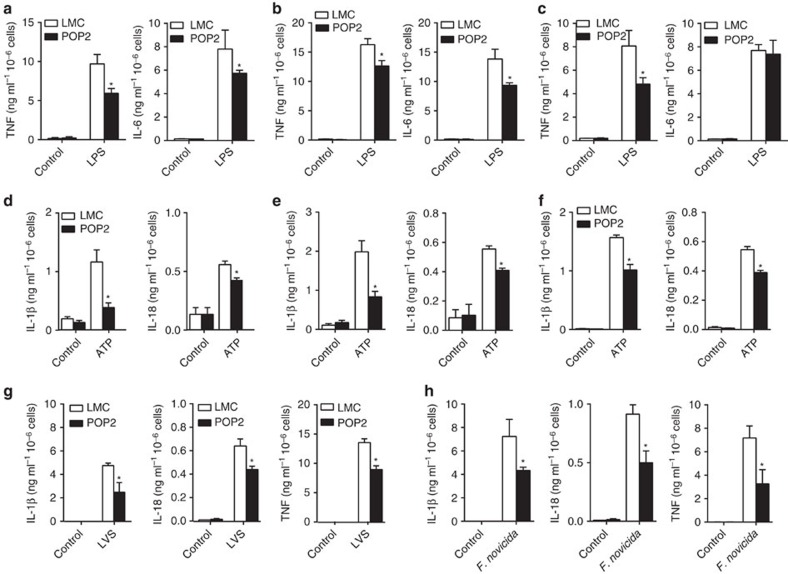
POP2 inhibits cytokine production in mouse macrophages. (**a**–**c**) Mean level±s.d. of TNF and IL-6 measured from culture supernatants of (**a**) BMDM, (**b**) peritoneal macrophages or (**c**) splenic macrophages from POP2Tg (*n*=4) and LMC (*n*=4) mice stimulated with LPS (100 ng ml^−1^) for 24 h. (**d**–**f**) Mean level±s.d. of IL-1β and IL-18 measured from culture supernatants of (**d**) BMDM, (**e**) peritoneal macrophages or (**f**) splenic macrophages from POP2Tg (*n*=4) and LMC (*n*=4) mice stimulated with LPS (100 ng ml^−1^) for 24 h plus ATP (5 mM) for the last 30 min. (**g**,**h**) Mean level±s.d. of IL-1β, IL-18 and TNF measured from culture supernatants of BMDM from POP2Tg (*n*=4) and LMC (*n*=4) mice infected for 24 h with (**g**) *F. tularensis* LVS (MOI=100) or (**h**) *F. novicida* (MOI=100). To determine significance, Student’s *t*-test was used. *P<0.05.

Consistent with NF-κB inhibition, LPS-induced TNF secretion was reduced in POP2 mouse macrophages (Fig. 2a–c). However, while LPS-stimulated BMDM and PM from POP2 mice produced less IL-6 (Fig. 2a,b), no difference in IL-6 secretion was observed between POP2 and LMC splenic macrophages (Fig. 2c). Regardless of their site of origin, POP2 mouse macrophages produced significantly less ATP-elicited IL-1β and IL-18 than those from LMC mice (Fig. 2d–f), reflecting regulation of inflammasome activation. However, while ATP-elicited IL-1β from POP2 mouse BMDM and PM was decreased by more than 50% (Fig. 2d,e), splenic macrophage was less profoundly affected (Fig. 2f). ATP-induced IL-18 responses were ∼25% lower in POP2 macrophages of all types. Curiously, IL-18 production was less affected by the presence of POP2 than IL-1β in both BMDM and PM.

The LPS of *Francisella tularensis* (*F. tularensis*) is non-stimulatory for TLR4 and TLR sensing of *F. tularensis* relies exclusively on recognition of bacterial lipoproteins by TLR2 (refs 21, 22, 23). As with LPS stimulation, POP2 BMDM infected with the *F. tularensis* live vaccine strain (*Ft* LVS) produced less IL-1β, IL-18 and TNF (Fig. 2g), thus POP2 regulation of macrophage inflammatory responses is not specific to TLR4/LPS. The precise TLR usage of *Francisella novicida* is not known; however, the inflammasome response to this bacteria requires the dsDNA sensor Aim2 instead of Nlrp3 (refs 24, 25). Importantly, in addition to diminished TNF secretion, POP2 BMDM infected with *F. novicida* produced significantly less IL-1β and IL-18 (Fig. 2h). This result confirms *in vitro* studies suggesting that POP2 likely regulates ASC-dependent inflammasomes other than Nlrp3 and provides initial evidence for POP2 regulation of the Aim2 inflammasome.

Collectively, POP2 regulates TLR and inflammasome-mediated macrophage inflammatory cytokine responses, consistent with our *in vitro* findings using mouse and human cells. However, this extent of this regulation varies by macrophage subset and type of inflammatory stimuli, demonstrating that regulation of inflammatory responses by POP2 is likely more complex than suggested previously.

### POP2 regulates the AIM2 inflammasome

**Figure 3:**
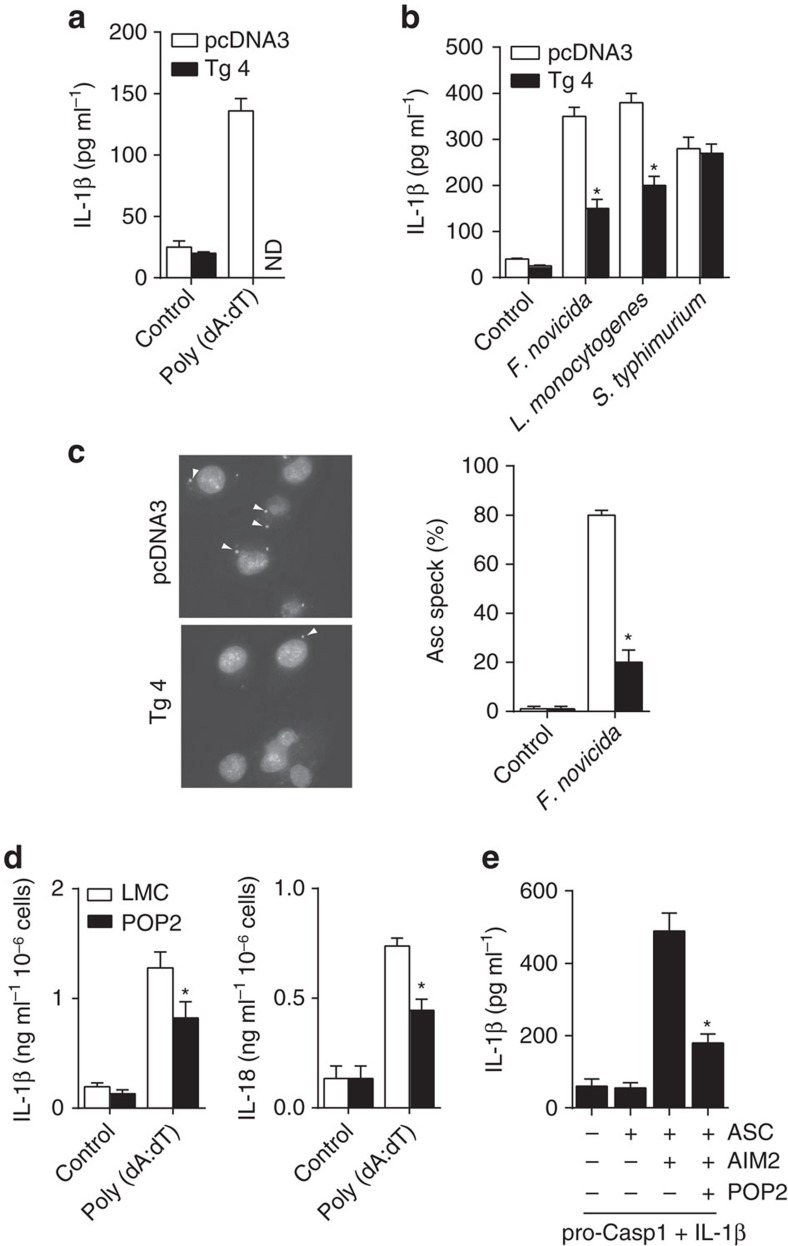
POP2 regulates the AIM2 inflammasome. (**a**) Mean level±s.d. of mature IL-1β measured from culture supernatants of pcDNA3 or POP2Tg J774A.1 transfectants treated with poly (dA:dT) (1 μg ml^−1^) for 18 h or (**b**) infected with *F. novicida* (MOI=100 for 24 h), *L. monocytogenes* (MOI=5 for 6 h) or *S. Typhimurium* (MOI=10 for 6 h) (*n*=3). (**c**) Immunohistochemical detection of Asc-speck formation (indicated by arrows) and per cent Asc specks in J774A.1 transfectants infected with *F. novicida* (MOI=100). (*n*>100 cells) (**d**) Mean level±s.d. of IL-1β and IL-18 measured from culture supernatants of BMDM from POP2Tg (*n*=4) and LMC (*n*=4) mice treated with poly (dA:dT) (1 μg ml^−1^) for 18 h. (**e**) Mean level±s.d. of mature IL-1β measured from culture supernatants of HEK293T cells transiently transfected with human ASC, AIM2 and/or POP2 constructs for 20 h (*n*=3). To determine significance, Student’s *t*-test was used. *P<0.05, ND, none detected.

Our findings suggest that in addition to the Nlrp3 inflammasome, POP2 also regulates the Aim2 inflammasome. Consistent with this hypothesis, stimulation of LPS-primed J774A.1 transfectants with the Aim2 inflammasome agonist poly(dA:dT) was blunted in the presence of the POP2 transgene (Fig. 3a). While the inflammasome response of macrophages to *F. novicida* is exclusively mediated by Aim2, *Listeria monocytogenes* activates both Nlrp3 and Aim2 inflammasomes^26^. By contrast, *Salmonella* Typhimurium, activates the ASC-independent Nlrc4 inflammasome^27^. Consistent with regulation of the Aim2 inflammasome by POP2, IL-1β production by POP2 transgenic J774A.1 cells was reduced after infection with *F. novicida* and *L. monocytogenes*, but not after *Salmonella* Typhimurium infection (Fig. 3b). Further, analysis of ASC oligomerization following *F. novicida* infection identified multiple ‘specks’ in J774A.1 control cells, which were dramatically diminished in POP2 transfectants (Fig. 3c), suggesting that POP2 impairs Aim2 inflammasome complex formation, likely via blockade of PYD:PYD interactions between AIM2 and ASC. Finally, BMDM from POP2 mice also produced less IL-1β and IL-18 in response to poly(dA:dT) (Fig. 3d). As POP2 inhibition of Aim2 might be specific to mouse, we reconstituted the human AIM2 inflammasome in HEK293T cells with and without POP2. In the absence of POP2, IL-1β was produced on poly(dA:dT) stimulation, but IL-1β elaboration was markedly impaired in the presence of POP2 (Fig. 3e), indicating that POP2 regulation of the Aim2 inflammasome is not species specific. Thus, POP2 is expected to regulate both Aim2 and Nlrp3 inflammasome-dependent inflammatory responses *in vivo*.

### POP2 broadly tempers *in vivo* inflammatory responses

**Figure 4:**
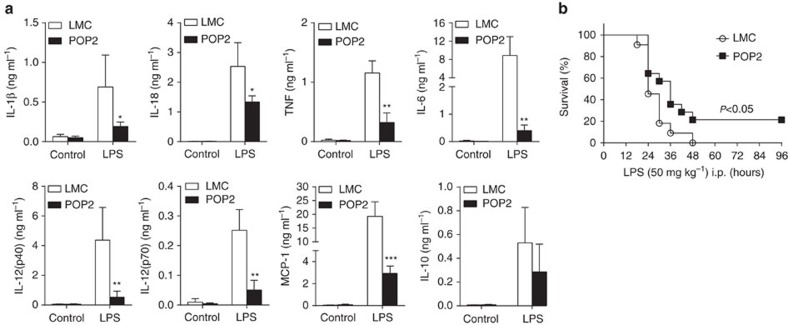
POP2 tempers the *in vivo* inflammatory cytokine response. (**a**) Mean serum levels±s.d. of IL-1β, IL-18, TNF, IL-6, IL-12p40, IL-12p70, MCP-1 and IL-10 in POP2 (*n*=4) and LMC (*n*=4) mice receiving a sublethal dose (15 mg kg^−1^) of LPS for 24 h by i.p. injection. To determine significance, Student’s *t*-test was used. (**b**) Survival of POP2 Tg (*n*=14) and LMC (*n*=14) mice after i.p. injection with a lethal dose (50 mg kg^−1^) of LPS. Data represent per cent of surviving mice from at least two independent experiments. To determine significance, Log-rank test was used. *P<0.05, **P<0.01, ***P<0.001.

Primary macrophage responses are unlikely to fully reflect the systemic impact of POP2 in response to *in vivo* inflammatory stimuli. Therefore, LMC and POP2 mice were intraperitoneally (i.p.) injected with a sublethal dose of LPS (*E. coli* O55:B5, 15 mg kg^−1^) and inflammasome- and NF-κB-dependent serum cytokines were measured. Although basal cytokine levels were essentially identical between unstimulated LMC and POP2 mice (Fig. 4a), POP2 mice had dramatically reduced levels of IL-1β, IL-18, TNF, IL-6, IL-12p40/70 and MCP-1 following LPS injection. With the exception of IL-18, these cytokines are NF-κB dependent, further suggesting that POP2 is broadly controlling NF-κB-dependent inflammatory cytokine expression. IL-10, which is less dependent on NF-κB in macrophages^28^,^29^ was not significantly diminished. Indeed, the absence of a marked IL-10 decrease in POP2 mice suggests that reduced production of IL-12 in these mice is not due to downregulation via IL-10 (ref. 30).

Certain cytokine gene-deficient mice are resistant to lethal LPS challenge^31^,^32^,^33^, including Nlrp3^−/−^ and Casp1/11^−/−^ mice, which are deficient in LPS-induced production of IL-1β and IL-18 (ref. 34). Consistent with the action of inflammatory cytokines in endotoxemia, 20% of POP2 mice survived an otherwise lethal dose of LPS (50 mg kg^−1^) and displayed a significant delay in time-to-death versus LMC mice (Fig. 4b). Thus, systemic reduction in pro-inflammatory cytokines by POP2 favours resistance to endotoxemia, but not to the same extent as IL-1β processing defects. Together, these data suggest that POP2 broadly dampens the systemic cytokine environment following inflammatory challenge and may limit the consequences of inflammation-associated pathology.

### POP2 improves resistance to bacterial infection

**Figure 5:**
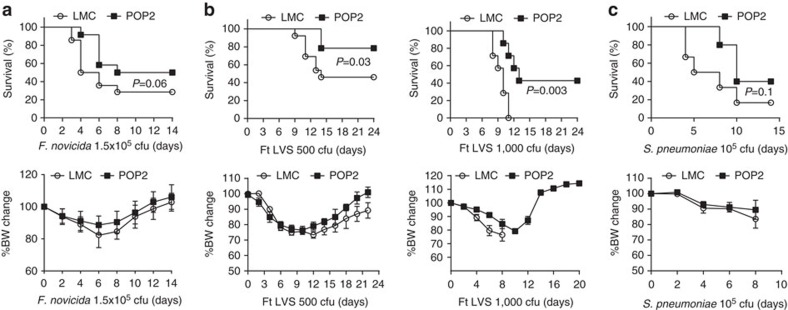
POP2 improves resistance to bacterial infections. (**a**–**c**) Per cent survival and mean body weight loss±s.d. in (**a**) POP2 (*n*=12) and LMC (*n*=14) mice after subcutanesous infection with 1.5 × 10^5^ c.f.u. of *F. novicida*, (**b**) POP2 (*n*=10) and LMC (*n*=10) mice after intranasal (i.n.) infection with 500 c.f.u. (LD50) of *F. tularensis* (left panel) and in POP2 (*n*=12) and LMC (*n*=12) mice after i.n. infection with 1,000 c.f.u. (lethal) of *F. tularensis* (right panel) and (**c**) POP2 (*n*=12) and LMC (*n*=12) mice after i.n. infection with 1 × 10^5^ c.f.u. of *S. pneumoniae*. Data represent per cent of surviving mice and accompanying weight loss from at least two independent experiments. To determine significance for survival data, the Log-rank test was used, and for weight data, Student’s *t*-test was used. *P* values are shown where significance was detected.

Inflammatory cytokine responses, including TNF, IL-1β, IL-6 and IL-12, mediate resistance to bacterial infection. For example, Aim2^−/−^ mice are highly susceptible to subcutaneous *F. novicida* infection, due to failed inflammasome activation^24^. Similarly, TLR2^−/−^ mice fail to mount TNF, IL-6 and IL-1β responses and are thus highly susceptible to *F. tularensis* pneumonia^23^. Thus, diminished inflammatory cytokine responses are expected to result in increased susceptibility to bacteria. By contrast, POP2 mice infected subcutaneously with *F. novicida* exhibited 20% greater survival, but little difference in weight loss (Fig. 5a). Most surprisingly, POP2 mice were significantly protected against pulmonary *Ft* LVS infection with 80% of the mice surviving an LD50 infection and ∼50% surviving a lethal infection (Fig. 5b). Weight loss of infected POP2 and control mice was comparable at both challenge doses.

As *Francisella* is a Gram-negative, intracellular pathogen, we considered whether POP2 mice might exhibit similar protection against the Gram positive, extracellular bacteria, *Streptococcus pneumoniae*. Following intranasal infection with 10^5^ c.f.u. of *S. pneumoniae*, POP2 mice were also resistant compared to controls (Fig. 5c). By contrast, mice infected with *Salmonella enterica* serovar Typhimurium via the orogastric route (10^6^ c.f.u.) had no reduction in bacterial burden and no alterations in a variety of relevant cytokine responses at 2 or 4 days post infection (Supplementary Fig. 6A–C), consistent with the lack of POP2 modulation of NLRC4 inflammasome responses seen *in vitro*. Collectively, instead of the universally diminished resistance to bacterial infection predicted by the broad moderating influence of POP2 on pro-inflammatory cytokines, resistance against certain bacterial infections is improved in POP2 mice. This surprising outcome suggests that POP2 may balance harmful inflammatory responses while promoting otherwise protective innate responses.

### POP2 reduces tissue damage and increases IFN-γ

**Figure 6:**
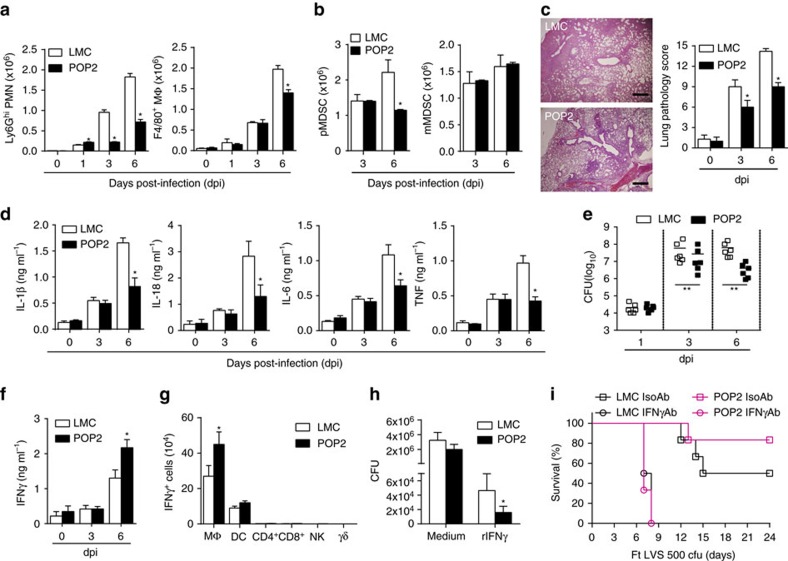
POP2 limits acute inflammation and increases IFN-γ in pulmonary tularaemia. (**a**) Mean total numbers±s.d. of Ly6G^+^ neutrophils and F4/80^+^ macrophages in lungs of POP2 (*n*=4) and LMC (*n*=4) mice infected with *F. tularensis* (*Ft*) LVS (MOI=100). (**b**) Mean total numbers±s.d. of polymorphonucleated-MDSC (pMDSC) and mono-nucleated-MDSC (mMDSC) in lungs of POP2 (*n*=4) and LMC (*n*=4) mice infected with *Ft* LVS (MOI=100). (**c**) Representative histologic lung sections from mice infected with *Ft* LVS showing inflammatory foci and necrosis (‘necrotizing inflammation’) at 6 dpi in LMC mice (top panel) and POP2 mice (bottom panel) (haematoxylin and eosin; Scale bar, 100 μm). Lung pathology scores for LMC (*n*=6) and POP2 (*n*=6) mice infected with *Ft* LVS (1,000 c.f.u.; LD100) (right graph) were calculated by analysis of lung sections for location, type and extent of inflammation and necrosis (see ‘Methods’ section). (**d**–**g**) POP2 (*n*=6) and LMC (*n*=6) mice were infected with *Ft* LVS (1,000 c.f.u.; LD100) and shown are (**d**) mean levels±s.d. of IL-1β, IL-18, IL-6 and TNF from lung homogenates, (**e**) bacterial burden in lungs, (**f**) mean levels±s.d. of IFN-γ measured from lung homogenates and (**g**) mean total numbers±s.d. of IFN-γ^+^ immune cells in lungs. (**h**) Mean bacterial count (c.f.u.)±s.d. from POP2 (*n*=4) and LMC (*n*=4) BMDM infected with *Ft* LVS (MOI=100) with and without administration of recombinant IFN-γ (100 IU). (**i**) Per cent survival of POP2 (*n*=12) and LMC (*n*=12) mice treated with 300 μg anti-IFN-γ antibody (catalogue number BE0055, BioXCell) on days −1, 2 and 4 or isotype control (catalogue number BE0088, BioXCell) and infected with *Ft* LVS (500 c.f.u., LD50). Survival of infected POP2 mice receiving isotype control differs significantly from that of POP2 mice receiving anti-IFN-γ (*P*<0.001) To determine significance, for **a**,**b**,**d** and **h**, Student’s *t*-test was used (*P<0.05); for **c**, scores were evaluated using the Mann–Whitney test (*P<0.05); for **e**–**g**, Student’s *t*-test was used (*P<0.05, **P<0.01), and for **i**, the Log-rank test, was used.

Ensuring neutrophil effector function during infection while avoiding tissue damage requires rigorous control^35^. During the course of a sublethal *Ft* LVS infection in mice, mature myeloid cells are needed to control the infection^36^. However, for lethal pulmonary *Ft* LVS infection, significant recruitment of immature myeloid/myeloid-derived suppressor cell (MDSC) cells and polymorphonuclear cells (PMNs) to inflammatory foci in the lung leads to dramatic lung pathology including hyaline membrane-like changes in alveolar epithelium, necrosis and host mortality^36^. Conversely, in the lungs of lethally infected POP2 mice, mature neutrophils (CD11b^+^ Ly6Ghi) or mature macrophages (CD11b^+^ F4/80^+^) numbers were not increased above control levels (Fig. 6a). Instead, neutrophils were reduced from day 3 forward. Numbers of immature granulocytes (PMN–MDSC) were also reduced at 6 days post infection, while immature monocytic cell (M-MDSC) numbers were unchanged (Fig. 6b). Despite the relative paucity of mature neutrophils and maintained numbers of MDSCs associated with lung damage and mortality, POP2 mice still survive *Ft* LVS infection. Moreover, POP2 mice also had significantly reduced lung pathology at 3 and 6 days post-*Ft* LVS infection (Fig. 6c). Further, lung concentrations of IL-1β, IL-18, IL-6 and TNF, cytokines associated with *Ft* LVS resistance^37^,^38^,^39^, were similar in POP2 and LMC mice during early infection (3 dpi) (Fig. 6d). However, at 6 dpi, these cytokines were all significantly reduced in POP2 mouse lungs and in serum (Fig. 6d and Supplementary Fig. 7). Despite dramatically lower neutrophil numbers and reduced cytokine levels, lung bacterial burden was significantly decreased in POP2 mice by 3 days post infection (Fig. 6e).

Thus, POP2 reduces the number of inflammatory cells appearing in the lung, which may account for significantly diminished inflammation-associated lung tissue damage while concomitantly improving bacterial control and overall host resistance. However, control of lung bacteria occurs in POP2 mice despite lower neutrophil numbers and all of these changes are evident as soon as 3 days before significant reduction in the lung levels of key inflammatory cytokines. Further, in contrast to resistance seen with wild-type mice at an LD50 or following elicitation of mature myeloid cells^36^, resistance of POP2 mice to *Ft* LVS occurs without an increase in mature myeloid cell numbers.

To address how POP2 establishes control of infection while simultaneously encouraging a less inflammatory environment, we considered the potential contribution of IFNγ. IFN-γ enhances macrophage killing and clearance of intracellular pathogens, including *F. tularensis*^40^,^41^,^42^. Moreover, mice deficient in IFN-γ or depleted of IFN-γ succumb more rapidly to pulmonary *Ft* LVS infection^43^, while IL-12 induction of IFN-γ is protective^44^. In contrast to diminished pro-inflammatory cytokines, IFN-γ was increased in the lungs of POP2 mice at 6 days post-*Ft* LVS infection (Fig. 6f). T cells (both CD4 and CD8), NK cells and γδT cells are major producers of IFN-γ although macrophages and dendritic cells can also make IFN-γ^45^. Surprisingly, IFN-γ producing macrophages were present in the lungs of *F. tularensis*-infected mice and increased approximately twofold in POP2 mice, whereas other major IFN-γ-producing cells were limited in number (Fig. 6g and Supplementary Fig. 8). Although macrophage numbers are lower in POP2 mice at day 6, the IFN-γ^+^ fraction (∼30%) is increased compared to wild type (∼12%). Numbers of IFN-γ-expressing DCs were unchanged. The ability of POP2 BMDMs infected with *Ft* LVS to control bacteria was examined. Bacterial numbers recovered from infected cells at 24 h post infection were comparable between untreated control and POP2 BMDMs (Fig. 6h). However, after treatment with recombinant IFN-γ, bacterial numbers were significantly lower in POP2 BMDMs, suggesting that POP2 may enhance the IFN-γ responsiveness of macrophages. Finally, while 80% of POP2 mice receiving isotype control antibody survive an LD50 infection with *Ft* LVS, survival was completely reversed by treatment with the IFN-γ-neutralizing antibody XMG.1 (Fig. 6i). Together, these results suggest that POP2 improves survival in an IFN-γ-dependent fashion, likely through improving lung macrophage production of IFN-γ and increasing the capacity of macrophages to kill or restrain the replication of bacteria. Thus, despite POP2-dependent tempering of lung and systemic inflammatory cytokine and cellular responses, POP2 also increases IFN-γ production in the lung and is likely responsible for more effective control of bacteria, which together improves host resistance.

Collectively, POP2 likely contributes significantly to the temporal modulation of immune cell recruitment, maturation and regulation of inflammatory cytokines during infection, potentially accounting for enhanced resistance to infection and protection from the deleterious effects of advanced inflammation on tissues. Further, in contrast to other POPs, POP2 either directly or indirectly controls the expression of IFN-γ, through mechanisms that are likely distinct from regulation of NF-κB signalling and inflammasome assembly/activation and which will require further study.

## Discussion

POP2 is a primate-restricted protein that inhibits NF-κB signalling and NLRP3 inflammasome activation *in vitro*, and implicated in controlling macrophage inflammatory cytokine responses^7^,^9^. However, POP2 regulation of inflammatory responses *in vivo* and how such regulation influences pathologic and/or beneficial aspects of inflammation has not been demonstrated. Using mice-expressing human POP2, this study confirms the anticipated *in vivo* functions of POP2, that NF-κB and Nlrp3 inflammasome-dependent cytokine responses to inflammatory challenge and infection are tempered by POP2. Despite this reduction in inflammatory cytokines important for innate immunity, POP2 mice were significantly more resistant to lethal bacterial infections. Mechanistically, POP2 enhances production of IFN-γ improving macrophage anti-bacterial function while concomitantly limiting inflammatory cytokine levels and reducing cellular inflammation, which promotes calculably diminished inflammation-derived tissue damage. Thus, although POP2 is an important modifier of inflammatory cytokine responses, it also enhances innate immunity, effectively serving to balance detrimental and beneficial aspects of the inflammatory response. We also provide insight into regulation of POP2 expression by identifying the ‘minimal’ promoter region and a 3′ARE controlling mRNA stability. Further, we reveal that POP2 regulates the AIM2 inflammasome *in vivo* and in human cells.

*POP2* expression is controlled by a promoter likely contained within 300 bp upstream of its translation start site. Short promoters are common in newly emergent genes^46^. Indeed, the promoter region of *NLRP2*, the parent gene of *POP2*, has a sizeable promoter^47^, but these sequences are absent upstream of *POP2*. Sequence analysis did not identify a transcriptional start site, suggesting *POP2* may have a dispersed/unfocused promoter recognized by RNA Pol II (ref. 48). The potential lack of a focused promoter is unsurprising as such promoters are more archaic and common in early eukaryotes, but less frequent among vertebrate genes^48^. However, a potential TFIIB-binding site (TATA box), a common feature of more ancient focused promoters^48^, and two initiator elements representing possible binding sites for TFIID^49^ are present. Consistently, only 127nt of the *POP2* 5′UTR appears to be required for NF-κB-mediated expression. The TATA box is also important and likely required for *POP2* transcription as NF-κB/p65 associates with TATA box-binding proteins TFIIB and TBP^49^. Further, *POP2* mRNA is rapidly induced by inflammatory stimuli^9^ and like TNF mRNA^50^ has a short half-life. As with other rapidly induced NF-κB-responsive genes, *POP2* has a 3′ARE regulating mRNA stability/degradation^50^,^51^. However, mutation of this ARE element incompletely restored expression, thus additional factors may regulate *POP2* mRNA stability. Some possibilities include a non-consensus AU-rich region in the 3′UTR^52^ or destabilizing RNA-binding proteins^53^. As these regulatory features are essential for appropriate mRNA expression and kinetics potentially influencing the *in vivo* action of POP2, the upstream regulatory sequence elements necessary for NF-κB-driven POP2 expression and the entire *POP2* 3′UTR were both included in our transgene.

As with human cells^7^, POP2 is constitutively expressed in major myeloid- and lymphoid-lineage cells and induced in primary macrophages from our POP2 mice. However, while expressed in POP2 mouse spleen, *POP2* mRNA was not detected in commercially available samples of human spleen^7^. Rapid decay of *POP2* mRNA owing to source, timing and method of organ harvesting, tissue integrity or stimulation might explain this discrepancy. Naive POP2 mice are healthy and generally indistinguishable from their non-transgenic littermates physically and haematologically, except for increased splenic neutrophil numbers. This increase could result from spleen-specific myelopoiesis, prevention of splenic neutrophil death or favoured development of the neutrophil lineage. However, as myelopoiesis is promoted via the interaction of c/EBP with NF-κB^54^,^55^, increased splenic neutrophil numbers may be unrelated to NF-κB inhibition by POP2. Further, it remains unclear why more neutrophils are not evident in blood or lymph nodes. Concurrent with this report, mice transgenic for CD68 promoter-drive POP2 targeting constitutive macrophage-lineage expression of POP2 have also been described^56^. The distinctions between constitutive macrophage-lineage specific and endogenously regulated POP2 expression are likely to prove useful in exploring the functions of POP2.

POP2 inhibits the transcriptional activity of NF-κB p65/RelA^7^,^9^,^13^ and impairs ASC:NLRP3 interaction^6^,^7^,^9^. POP2 also inhibits activation of both the mouse and human AIM2 inflammasome (this report). Consequently, the reduced NF-κB- and Nlrp3 inflammasome-dependent cytokine responses of POP2 mouse macrophages were anticipated. These findings are similar to those observed using CD68-POP2 mice^56^. However, TNF, IL-6, IL-1β and IL-18 levels differ in POP2 macrophages from different lymphoid tissues. The reason for this is not clear, nor has an exhaustive survey of cytokines produced by these cells been completed. Further, POP2 modulation of cytokines differs depending on the inflammatory stimuli (that is, LPS, ATP or active infection). While intriguing, these disparate results attest that POP2 does not promote homogenous responses among cells of the innate immune system and may confer heterogeneity. Indeed, POP2 alterations of cell type and cytokine responses may promote optimal tissue-specific immune responses to ensure clearance of varying insults without added injury.

Although inflammatory cytokines important for innate immunity were broadly and markedly reduced *in vivo*, POP2 mice were better protected against lethal bacterial pneumonia caused by *F. tularensis* or *S. pneumoniae*. In the *F. tularensis* model, prominent lung infiltration by immature myeloid cells/MDSC with poor phagocytic capacity drives host mortality^36^ but mature neutrophils are important for resistance^36^,^57^. In seemingly stark contrast, significant bacterial control is evident by day 3 in POP2 mice and lung damage is constrained, but with fewer mature neutrophils and without reduction in immature cell numbers until day 6. Enhanced production of IFN-γ in POP2 mice, with a notable increase in IFN-γ-producing lung macrophages and improved macrophage responsiveness to IFN-γ, is essential. POP2 is thus tempering pro-inflammatory cytokine and neutrophil responses while improving the host capacity to control infection and limit damage. While improvement of macrophage function due to IFN-γ seems the most likely explanation for controlling bacterial numbers, our data do not exclude the possibility that POP2 might also directly or indirectly regulate production of antimicrobial chemokines such as CXCL10 (refs 58, 59). This homoeostatic balancing of responses is intriguing and points to a broader than anticipated contribution for POP2. Detailed attention to the mechanism(s) responsible is required.

IFN-γ contributes to homoeostasis in part by attenuating tissue infiltration of inflammatory sites by neutrophils and monocytes^60^, a potential explanation connecting increased IFN-γ in infected POP2 mice with diminished neutrophil and pMDSC numbers. IFN-γ-mediated changes in chemoattractant expression including MCP-2 (ref. 61), responsiveness to chemoattractants^62^ and attenuation of granulopoiesis along with improved monopoiesis during infection^63^ may all contribute. Consistent with the homoeostatic impact of IFN-γ, serum levels of the chemokines KC/Cxcl8 and MCP-1 are also lower in infected POP2 mice, although this may be attributed to NF-κB inhibition. Curiously, reduced numbers of neutrophils and monocytes are elicited in CD68-POP2 mice after i.p. introduction of monosodium urate, although this is attributed to reduced IL-1β production^56^. Accelerated myelopoiesis/emergency granulopoiesis is an important feature of various bacterial infections including pulmonary tularaemia^36^,^64^. In addition to limiting myeloid cell infiltration, IFN-γ attenuates tissue damage mediated by these cells by reducing expression of multiple matrix metalloproteases, serine proteases and other proteases in part through suppressing IL-1R expression^59^. Reduced production of IL-1β in POP2 mice may also limit detrimental IL-1R-mediated signals. It will be of considerable interest to further dissect how POP2 influences IFN-γ homoeostatic and tissue protective effects.

Immature myeloid cells/MDSC contribute to host mortality in *F. tularensis* bacterial pneumonia. Importantly, MDSC from infected wild-type mice do not mature further *ex vivo* and are poorly phagocytic^36^. PMN–MDSC are much less numerous on day 6, but at this point, inflammatory mediators are also reduced and bacterial control has been ongoing for 3 days. Granulocytic cells have shorter half-lives than monocytic cells and the decline in PMN–MDSC is more consistent with the initiation of homoeostatic processes as the infection resolves (reduced granulopoiesis and contraction of existing cells). From this perspective, it seems likely that the decline in pMDSC is not reflective of a protective mechanism. We do, however, see an increase in mature neutrophils at day 6, although of much lower magnitude than in wild type, so we cannot completely rule out possibly improved maturation of immature neutrophils.

Maturation and functionality of the lung myeloid compartment is important for protection, tissue integrity and survival. Neutrophils utilize degranulation, phagocytosis and production of neutrophil extracellular traps to control initial infection^65^. Yet, strict coordination of cell numbers, phenotype and optimal function is required to clear bacteria without excessive recruitment leading to tissue damage. Functionally, POP2 may influence the migration and the balance between mature and immature neutrophils. In *F. tularensis*-infected POP2 mice, the mature neutrophil response is enhanced within the first 24 h of infection. Although the numbers of neutrophils are only slightly higher, this may explain reduced bacterial burden at day 3. Further, neutrophil maturation is extremely important during sepsis or severe inflammation, as inadequate migration, phagosomal acidification and increased prevalence of immature neutrophils results in rapid death of both mice and humans^66^. Thus, reduced lung damage in POP2 mice may result, in part, from fewer immature granulocytes, although the number of phenotypically immature neutrophils (PMN–MDSC) persists until around day 6. As a primary reservoir for many intracellular pathogens, macrophage function is also important. While we do not see any meaningful change in the numbers of mature macrophages until day 6 when they are less abundant, our data suggests that IFN-γ-induced control of bacterial numbers is enhanced in POP2 mouse macrophages. Moreover, the phagocytic and bacteriocidal/bacteriostatic properties of both neutrophils and macrophages are increased by IFN-γ (refs 67, 68). Finally, the number of IFN-γ-producing macrophages is higher in the lungs of *F. tularensis*-infected POP2 mice. Collectively, our findings illustrate that via increasing IFN-γ, POP2 likely improves macrophage anti-bacterial functions while moderating the number of neutrophils, potentially reflecting a better balance of neutrophil effector function versus tissue damage.

The number of IFN-γ-expressing macrophages present in POP2 mice at day 6 in our infection model is substantial, while the number of IFN-γ^+^ CD4 and CD8 T cells, NK cells and γδT cells are low. Myeloid cells including macrophages and DCs produce IFN-γ, which contributes to intracellular host defence^69^,^70^. However, how POP2 promotes increased IFN-γ is unclear, especially in light of reduced IL-12 and IL-18. Several possibilities are worth consideration. First, myeloid expression of the major IFN-γ transcription factor T-bet is known^70^ and might be positively regulated by POP2. Second, macrophage STAT5 could be similarly involved^71^,^72^. In Nlrp3KO mice, the pro-Th2 transcriptional activity of Nlrp3 is absent and a pro-Th1 environment is favoured^73^, but it is not clear whether similar transcriptional changes might occur in myeloid populations in the lung or be regulated by POP2.

Collectively, the overall impact of POP2 on the inflammatory and innate response to bacteria appears complex and potentially multifactorial. Although limiting pro-inflammatory cytokines as expected of a bona fide regulator of inflammatory processes, POP2 also acts in an unanticipated fashion to provide homoeostatic balance during inflammatory challenge and/or infection in part via increasing IFN-γ by an unknown mechanism. In addition, this homoeostatic balance might also be influenced by modulation of NLR/inflammasome function. For example, studies of POP2 interactions with PYDs suggest that ASC-dependent inflammasomes might be generally inhibited by POP2 (refs 6, 7). Consistent with these *in vitro* studies, Nlrp1b and Nlrp7 inflammasome activation was impaired in BMDM from transgenic mice with macrophage-lineage-restricted expression of POP2 (ref. 56). However, whether POP2 regulates inflammasomes involved in homoeostasis of gut mucosa and microbiota (for example, NLRP6 and NLRP12)^74^,^75^ and whether they might impact similar processes in the lung is unknown. Thus, POP2 is anticipated to exert a variety of important functions impacting numerous infectious diseases and inflammatory conditions, highlighting the utility of our POP2 transgenic mouse model for better understanding the function of this human protein. Further, functional polymorphisms in POP2 could potentially tilt the scales favouring increased inflammation and/or diminished homoeostatic control with significant implications for the pathogenesis and potential treatment of immune diseases.

## Methods

### Generation of POP2 transgene construct

POP2 promoter regions were amplified using site-specific primers with added KpnI and HindIII sites and cloned into pGL3 (Promega, Madison, WI) upstream of the luciferase gene. Primers are as follows: hPop2promoter-F-2000, bp KpnI 5′-aa ggt acc gca tgc cat agg aat tta-3′; F505 bp KpnI 5′-aa ggt acc CAG AAG AAT AAA GTT GGA CC-3′; F250 bp KpnI 5′-aa ggt acc ATA AGA CAA CTC ACA GAA TGG-3′; F128 bp KpnI 5′-aa ggt acc AAT GAG CAA AAA TCT GAA CTG-3′ and hPop2promoter-rev-HindIII 5′-Tt aag ctt ctt gtc cta tgt ggg agc-3′. The 3′-UTR of POP2 (∼240 bp region) was cloned downstream of the pGL3 luciferase gene using the *XbaI* site (non-directional cloning) using pcDNA3 expressing POP2 transgene as template, and the following primer sequences, forward: 5′-AAA TCTAGA CCC CTC AGG GAT AGT GAG-3′; reverse: 5′-AAA TCTAGA AGC CTA GTG GAT ATG AAG TG-3′. Mutation of the ATTTTA hexamer (ARE element) in pGL3-POP2-3′UTR using the QuikChange-site directed mutagenesis kit (catalogue number 200523, Stratagene) and the following primers, forward: 5′-AGC CTT GGT TCT GCC TCC ATAATA CAT GCA CAT GTT GCT TA-3′; reverse: 5′-TAA GCA ACA TGT GCA TG TATTAT GG AGG CAG AAC CAA GGC T-3′.

### Generation of J774A.1 macrophages expressing POP2 transgene

The pcDNA3-HA-POP2 Tg vector was digested with BglII and HindIII restriction enzymes to remove the CMV promoter of the pcDNA3 plasmid (Invitrogen), and purified after agarose gel electrophoresis using Qiaex II gel extraction kit (catalogue number 20051, Qiagen). J774A.1 (5 × 10^6^ cells) were suspended in 500 μl serum-free DMEM and were electroporated with 10 μg of purified linear pcDNA3 vector encoding POP2 transgene using a GenePulser (BioRad; 570 V, 25 μF, time constant ∼0.9). Cells were transferred immediately to a 150 mm Petri dish containing complete culture medium. After 2 days, transfected cells were selected using 1 mg ml^−1^ G418 (Invitrogen) with a complete change of culture media every 2–3 days for 2 weeks.

### Cell culture and stimuli

HEK293T, HeLa and the mouse macrophage J774A.1 cell lines were cultured in DMEM (with 4.5 g liter^−1^ glucose) supplemented with 10% heat-inactivated foetal bovine serum, 5 mM L-glutamine and 0.1% penicillin–streptomycin (complete culture media). Human monocytic THP-1 and U937 cell lines were cultured in RMPI-1640 medium supplemented as above. All the cell lines were obtained from American Type Culture Collection (ATCC).

Ultrapure *E. coli serotype* O26:B6 LPS (Sigma), nigericin (Invivogen), ATP (Invivogen), and poly (dA:dT) (Invivogen) were used for activation of NF-κB and inflammasomes pathways. For *in vitro* stimulation, cells cultured in 24-well plates (2.5 × 10^5^ cells per well) were stimulated with LPS (100 ng ml^−1^), ATP (5 mM ml^−1^), nigericin (10 μM0 or poly (dA:dT) (1 or 5 μg) for indicated time points as described in figure legends. For analysing the half-life and stability of POP2 mRNA, LPS-treated THP-1 cells were further treated with ActD (5 μg ml^−1^; Sigma-Aldrich) and qRT–PCR analysis was performed. In other experiments, THP-1 and U937 cells were treated with cycloheximide (10 μg ml^−1^; Sigma) for 6 h and qRT–PCR was performed.

For bacterial infection, J774A.1 cells (2.5 × 10^5^ per well) expressing pcDNA3 control vector or POP2 transgene were cultured in antibiotic-free media and were infected with *Salmonella enterica* serovar *typhimurium* (NCIMB 13284; MOI=10), *Listeria monocytogenes* (NCIMB 13726; MOI=5) or *Franciscella novicida* (U112 strain; MOI=100). *S. typhimurium* and *L. monocytogenes* were grown in tryptic soy agar, while *F. novicida* was grown in chocolate agar and later subcultured in brain heart infusion broth. Cultured bacteria were aliquoted and stored frozen at −80 °C. The viable bacterial numbers (c.f.u.) in frozen vials were calculated by spread-plate method. At the time of infection, frozen vials of bacteria were thawed and used for infection according to the indicated multiplicity of infection (MOI).

### Generation of POP2 transgenic mouse

The transgenic founder mouse was generated at the Center for Functional Genomics, State University of New York, Albany, NY by pronuclear microinjection of POP2 Tg construct (shown in Supplementary Fig. 2A) into fertilized oocytes/eggs obtained a C57BL/6 X BALB/c cross. POP2 transgenic mouse generated initially on a BALB/c background were backcrossed onto the C57BL/6 background for at least nine generations. In all the experiments, littermates were used as controls. POP2 and LMC mice were housed in the specific pathogen free suite in the Animal Resources Facility at Albany Medical College. Food and water were provided *ad libitum*. All animal procedures were approved by the Albany Medical College Institutional Animal Care and Use Committee. All experiments were conducted using equal numbers of male and female mice of 6–9 weeks of age. POP2 transgenic neonates were free of birth defects and exhibited no obvious differences in general phenotype or pre- and post-weaning behaviours. Further, male and female POP2 mice are fertile with no apparent differences in age of sexual maturity. Litter size, body weight and sex distribution within litters and between LMC and POP2 mice were also unaffected. Vital organs (kidneys, spleen, heart, lungs, testes, bladder and liver) and lymphoid organs (spleen, thymus and lymph nodes) were normal in size, weight and gross anatomy with no appreciable changes in histology (Supplementary Fig. 9).

### Genotyping of transgenic mice

Genomic DNA isolated from ear punch were amplified in PCR using Premix Taq (TaKaRa Ex Taq Version 2.0) and 20 pmol of forward and reverse POP2 primers (5′-AAGGAGCTACAGACCGTCCC-3′ and 5′-TAAGGTGGGGGCATCACACA-3′) under the following PCR conditions: 5 min at 95 °C; 30 cycles of (30 s at 95 °C, 30 s at 60 °C and 1 min at 68 °C) and 2 min at 72 °C. The PCR products were run on a 1.5–2% agarose gel and 85 bp of the amplicon was size identified.

### Isolation and *in vitro* treatment of mouse macrophages

Bone marrow cells were isolated from the femurs and tibias of six- to eight-week-old mice and cultured in DMEM containing L-cell supernatant to enrich BMDM. Splenic macrophages were isolated by magnetic separation and cultured in DMEM. Peritoneal-resident macrophages were isolated by lavaging peritoneal cavity with 10 ml sterile cold-PBS and cells were cultured in DMEM. Macrophages were plated at 10^6^ cells per well in six-well plates and stimulated with LPS and cytokines were measured at indicated time points.

### Luciferase assay

A total of 2 × 10^5^ HEK293T cells per well were seeded in six-well plates and allowed to incubate overnight. For POP2 promoter truncation and TATA mutants, HEK293T cells were co-transfected with 500 ng of POP2 and either 500 ng of p65 or pcDNA3 control constructs at a 2.5:1 ratio of FuGENE 6 (Promega) to DNA (μg) and incubated for 24 h. Cells were lysed with reporter lysis 5 × buffer (catalogue number E397A, Promega) and detection of luciferase activity was measured using the Victor3V luminometer (Perkin Elmer Life Sciences and normalized to total protein. Similarly for the 3′ARE-luciferase assays, HeLa cells were transfected with 1 μg of indicated constructs in FuGENE 6 at a 3:1 ratio to DNA and luciferase activity was measured as above.

### mRNA stability and half-life studies

ActD (5 μg ml^−1^; Sigma-Aldrich) was added directly to THP-1 cells primed with LPS (100 ng ml^−1^) for 30 min. Cells were incubated for the indicated time after addition of ActD (time=0), followed by qRT–PCR analysis. Cycloheximide (10 μg ml^−1^; Sigma) was added directly to the THP-1 and U937 cells in culture and the cells were incubated for 6 h before RNA isolation

### Inflammasome reconstitution assay in 293T cell

Cells (5 × 10^4^ cells per well) were seeded in a 24-well plate, followed by transfection with plasmids encoding pro-caspase-1 (50 ng), pro-IL-1β (200 ng), ASC (20 ng) and AIM2 (100 ng) with or without POP2 (500 ng) using FuGENE 6 as per the manufacturer’s instructions (Promega). Production of IL-1β was measured by ELISA 18–24 h following stimulation.

### Speck formation assay

Cells (1 × 10^5^) were seeded in 0.5 ml antibiotic-free media in four-well poly-lysine-treated glass chamber slides (BD Biosciences). Cells were infected with *F. tularensis* U112 at MOI=100 for overnight and fixed in 4% paraformaldehyde. Cells were permeabilized in 0.1% Triton X-100 for 15 min and blocked with 10% goat serum. Cells were stained with 1:100 diluted rabbit anti-human ASC (Santa Cruz Biotech) and then with 1:1,000 diluted goat anti-rabbit-594 (Invitrogen) antibody. Slides were covered with glass coverslip using VectorShield mounting agent containing DAPI. Slides were imaged using in fluorescence microscope (Carl Zeiss). At least 50 cells were counted to determine the percentage of ASC-speck-containing cells.

### RT–PCR and quantitative RT–PCR

RNA was isolated using RNeasy purification columns (Qiagen) and treated with DNAase I. Quantitative PCR was performed using the SuperScript III Platinum SYBR Green One-Step qRT–PCR kit (catalogue number 11736-051, Invitrogen). All reactions were run in triplicate. *C*_t_ values were normalized to β-actin as an internal control and relative copy numbers calculated by the standard 2^−dCT^ method. Semi-quantitative RT–PCR was performed using ∼500 ng of RNA and One-Step RT–PCR Kit (catalogue number 210212, Qiagen) according to the manufacturer’s instructions and with primers published previously^9^. POP2 primers for both RT–PCR and qRT–PCR are the same as the genotyping primers described above. Mouse GAPDH RT–PCR primers for RT–PCR (452 bp) were forward: 5′-ACCACAGTCCATGCCATCA-3′ and reverse: 5′-TCCACCACCCTGTTGCTGT-3′. Human β-actin primers for qRT–PCR(103 bp) were forward: 5′-CCCCCATGCCATCCTGCGTCTG-3′ and reverse: 5′-CTCGGCCGTGGTGGTGAAGC-3′.

### LPS injection of mice

POP2 and LMC mice were i.p. injected with a single bolus of LPS (50 mg kg^−1^ body weight) and monitored every 12 h for clinical response and survival/mortality pattern up to 4 days. For serum cytokine analysis, POP2 and LMC mice were injected i.p. with LPS (15 mg kg^−1^ body weight) and blood was collected after 24 h by facial vein bleeding. The serum separated from the clotted blood was processed by luminex assay^36^.

### Bacterial infection of mice

For intranasal bacterial infection, mice were anaesthetized by a mixture of ketamine (20 mg ml^−1^) and xylazine (1 mg ml^−1^) and a 40 μl of bacterial suspension containing 5 × 10^2^ or 10^3^ c.f.u. of *Ft* LVS or 10^5^ c.f.u. of *S. pneumoniae* was instilled in one nare. For subcutaneous bacterial infection, 100 μl of bacterial suspension containing 1.5 × 10^5^ c.f.u. of *F. novicida* U112 was injected. For *Salmonella* Typhimurium (Thermo Fisher), mice were infected with 10^6^ c.f.u. by intragastric lavage. Infected mice were monitored for survival/mortality pattern or killed for tissue/cytokine analyses at specified days post infection. Blood was collected by submandibular venipuncture and serum was separated^36^. Killed mice were necropsied and spleen, liver and lungs were collected aseptically. The smaller lobe of the lung was used for preparation of lung homogenate for bacterial counting and/or cytokine assay and the remainder was used for isolation of single-cell suspensions for flow cytometry analysis of infiltrating cells. Remaining tissues were fixed in 10% buffered formalin for histology. *Francisella* species were provided by the Microbiology Core at the Albany Medical College Department of Immunology and Microbial Disease. *S. pneumoniae* was provided by Dr Dennis Metzger.

### Immunophenotyping by flow cytometry

For immunophenotyping of immune cells and other flow cytometry analysis, the following antibodies were used. Mouse PE-anti-CD3 (clone 17A2), AF647-anti-CD3e (clone 145-2C11), FITC-anti-CD4 (clone GK1.5), PE/Cy7-anti-CD4 (clone GK1.5), AF488-anti-CD8a (clone 53-6-7), PE-anti-NK1.1 (clone PK136), APC-anti-CD25 (3C7), PE-anti-FoxP3 (clone 150D), PE-anti-IgG1 isotype control (clone MOPC-21), APC-anti-IL17A (clone TC11-18H10.1), APC-anti- rat IgG1 isotype control (clone RTK2071), FITC-anti-CD11b (clone M1/70), PE/Cy7-anti-CD11b (clone M1/70), ACP-anti-CD11c (clone N418), APC/Cy7-anti-CD11c (clone N418), PB-anti-B220 (Clone RA3-6B2), AF700-anti-CD19 (clone 6D5), PE-anti-F4/80 (clone BM8), PB-anti-Gr-1 (clone RB6-8C5), PerCP.Cy5.5-anti-CD49 pan NK cell (clone DX5) and PE/Cy7 rat IgG2b isotype control antibodies were purchased from BioLegend. Immunophenotyping was performed by using antibodies against select surface markers specific for lymphoid and myeloid cells of spleen, thymus, pulmonary lymph nodes, peritoneal fluids, bone marrow cells and peripheral blood. Cells were incubated with appropriate antibodies for 30 min at 4 °C, washed and fixed in 1% paraformaldehyde. Isotype-matched controls were used in all phenotyping experiments^36^.

For detection of POP2 Tg expression by flow cytometry, we used anti-HA-tag antibody or POP2 antibody. J774 POP2 transgenic cells or single-cell suspensions obtained from spleen, bone marrow and peripheral blood of transgenic and LMC mice were surface stained with select markers for 30 min and fixed in 1% PFA and then permeabilized (0.5% Triton X-100 for 30 min at 4 °C) before staining for 40 min on ice with 1:400 diluted POP2 antibody (Santa Cruz) or 1:400 biotin-HA-tag antibody (Cell Signaling Technologies) and then 1:6,000 streptavidin-FITC (BioLegend) for 30 min at 4 °C. Multi-parameter FACS analysis was performed on a LSRII instrument (Becton Dickinson) and data were analysed using FlowJo software (v10.0.1; Tree Star). Specific cell populations are represented as a mean percentage of the cells from *F. tularensis*-infected mice at various dpi in comparison to uninfected control mice (0 dpi). In addition to percentage of specific cell types observed, the total numbers of cells were calculated as well^36^.

### Histology and immunohistochemistry

After necropsy, organs were collected for examination of gross pathology. A representative tissue sample from each organ was fixed in 10% buffered formalin for histology. In bacterial infection experiments, small pieces of lungs, spleen and liver were used for bacterial re-isolation and cytokine measurements. The formalin-fixed tissue samples were cut into pieces of 2–3 mm thickness and washed thoroughly with water for several hours before putting in ascending grades of alcohol for dehydration. The dehydrated tissues were cleared in turpentine oil and embedded in paraffin blocks. Sections of 4 μm thickness were cut from paraffin blocks. The sections were routinely stained with haematoxylin and eosin. The stained sections were examined under light microscope (Leitz). Sections of lungs, spleen or liver were examined for the location of inflammatory foci, type of infiltrating cells and the extent of necrosis and a pathology score was calculated using our established criteria (Supplementary Fig. 10).

### Cytokine assay

A panel of mouse and human cytokines were measured in culture supernatants and mouse serum or lung homogenates by Luminex assay (catalogue number 171304070M, Biorad) and/or ELISA (catalogue numbers 88-7064 (mouse IL-6), 88-7324-88 (mouse TNF) and 88-7013-88 (mouse IL-1β), eBioscience) using commercially available kits following the manufacturer’s instructions^36^.

### Cell death assays

Cell death analyses were performed by measuring LDH release (CytoTOX colorimetry, Promega) in culture supernatants and fluorescent staining of cells with DNA intercalating dyes (7-AAD or PI) for flow cytometry analysis following standard procedures^36^.

### Statistical analysis

For all *in vitro* studies, three independent experiments with at least two to three biological replicates per experimental group were performed, unless mentioned in the figure legends. Data were normalized to the standard or control as appropriate. For *in vivo* experiments, in general, groups of at four–six mice were evaluated and experiments were repeated two to three times as appropriate. Statistical analyses including Student’s *t*-test, log-rank test (Mantel–Cox), and Mann–Whitney used where indicated in the figure legends were performed using GraphPad Prism software (v6). Differences between control and experimental groups were considered significant at *P*< 0.05.

### Data availability

The authors declare that the data supporting the findings of this study are available within the article and its Supplementary Information files.

## Additional information

**How to cite this article:** Periasamy, S. *et al*. Pyrin-only protein 2 limits inflammation but improves protection against bacteria. *Nat. Commun.* **8,** 15564 doi: 10.1038/ncomms15564 (2017).

**Publisher’s note:** Springer Nature remains neutral with regard to jurisdictional claims in published maps and institutional affiliations.

## Supplementary Material

Supplementary Information Supplementary Figures
