# Supplementary material for: Pyrin-only protein 2 limits inflammation but improves protection against bacteria
Source: Nat Commun. 2017 Jun 5;8:15564. doi: 10.1038/ncomms15564 (PMC5512670; doi:10.1038/ncomms15564)
Supplement: Supplementary Information — Supplementary Figures [file ncomms15564-s1.pdf]

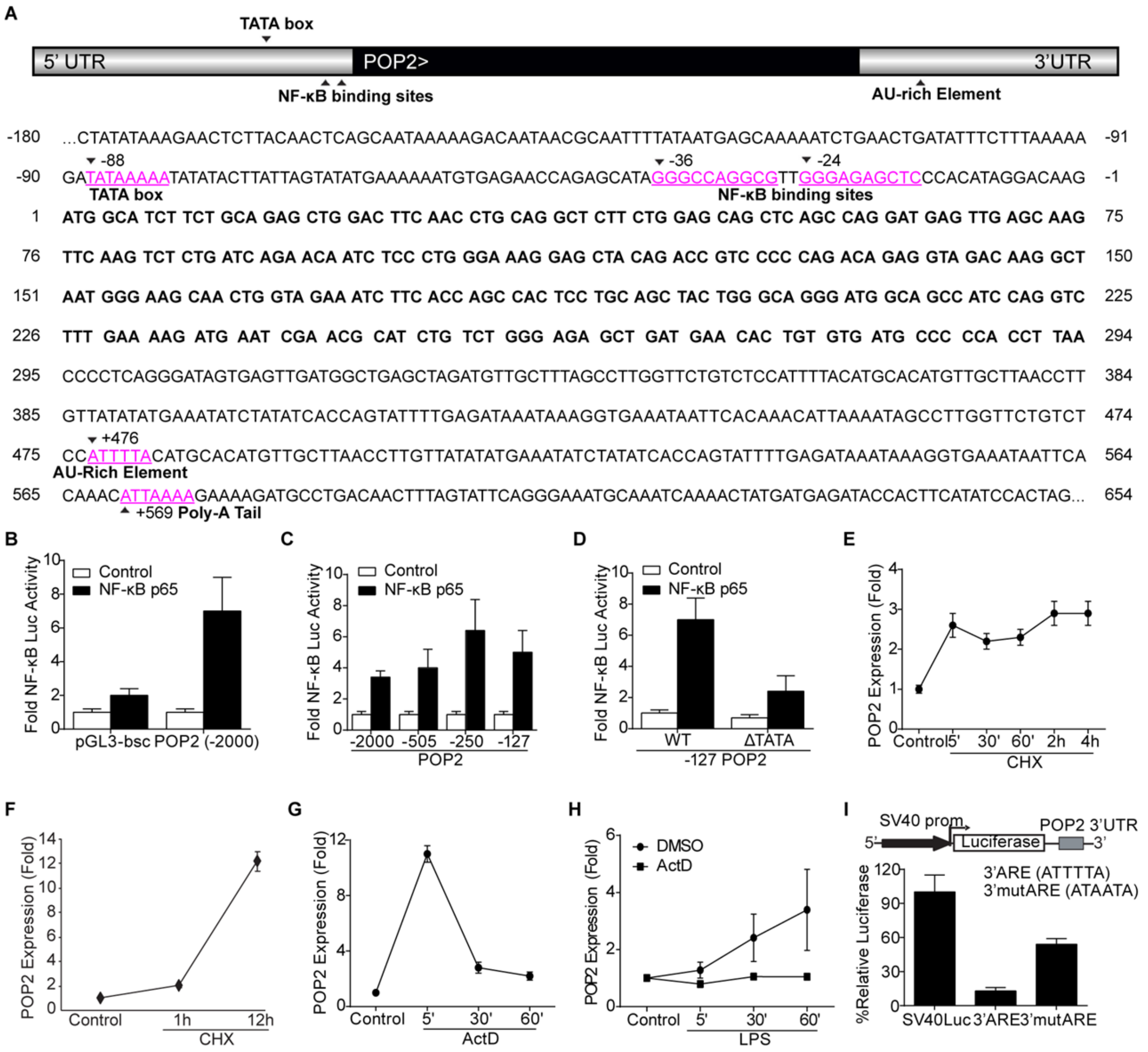

Supplementary Figure 1. **Characterization of the POP2 transcriptional and post-transcriptional regulatory elements.** (**A**) POP2 nucleotide sequence depicting the consensus sequence for the TATA box and NF-kB binding sites at upstream level (5'UTR), and the AU-rich element at 3'UTR region. **(B)** Luciferase assays following transfection of HEK-293T cells with constructs containing the -2000bp region upstream of the POP2 ATG and NF-κB p65 or control plasmid. **(C)** Luciferase assays using 5' truncations of the -2000 POP2 promoter with NF-κB p65 in HEK-293T cells. **(D)** Luciferase assays using constructs with WT and TATA-box mutations in the -127 POP2 promoter region in HEK-293T cells. **(E)** POP2 mRNA expression in LPS-primed THP-1 cells treated with cycloheximide (CHX). (**F**) POP2 mRNA expression in LPS-primed U937 cells treated with cycloheximide (CHX). **(G)** POP2 mRNA half-life was determined by inhibition of transcription with ActD following LPS stimulation. (**H**) ActD treatment prior to LPS priming completely inhibited POP2 mRNA expression. **(I)** A model for WT POP2 3'UTR or 3'ARE mutant constructs and luciferase assays in HeLa cells transfected with either WT POP2 3'UTR or 3'ARE mutant constructs. (B-I). Data in B-I are normalized to the appropriate controls and represent means ± SD from two or more independent experiments.

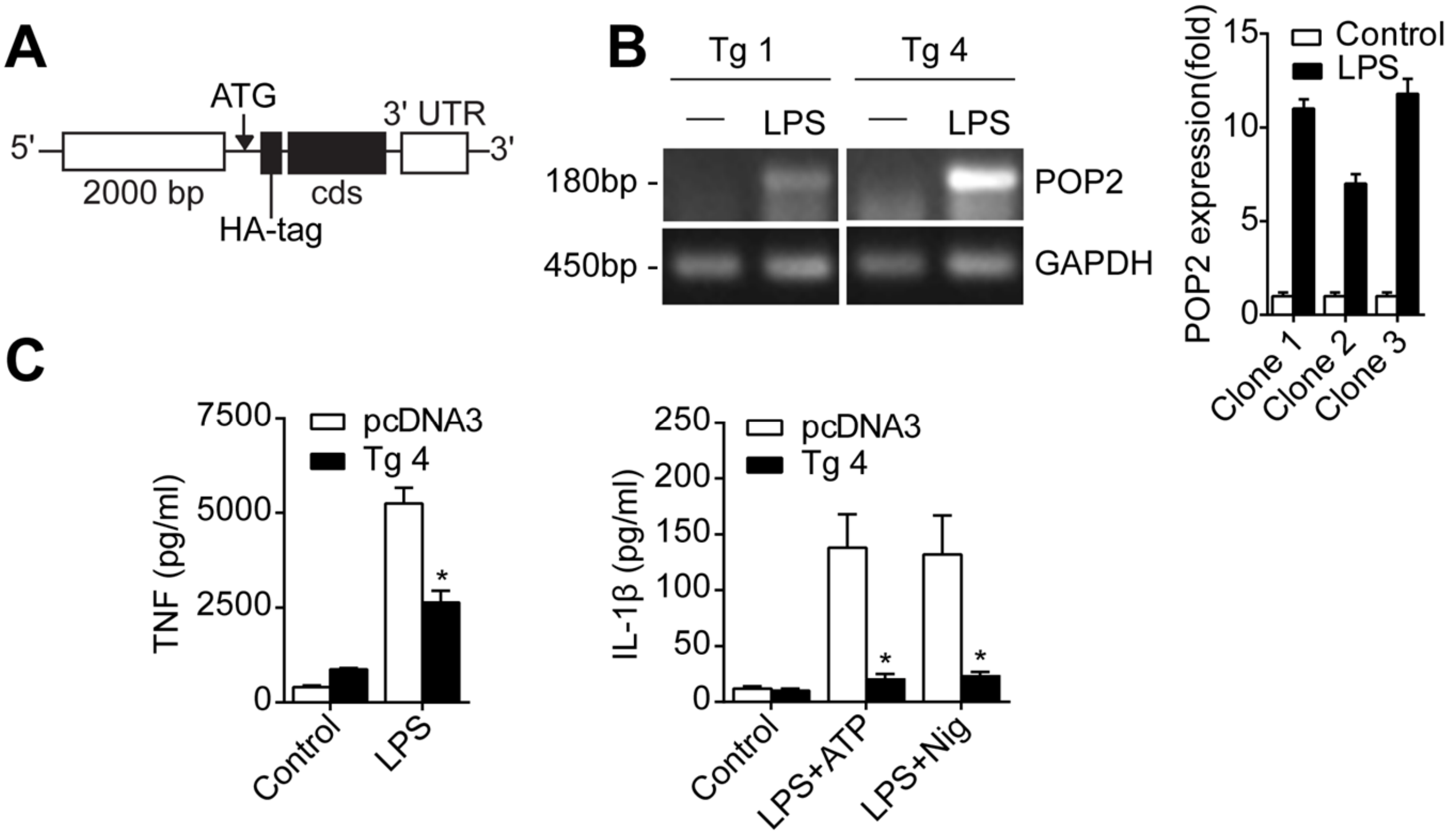

Supplementary Figure 2. **Generation and function of human POP2 Transgene. (A)** A model of the POP2 Tg construct containing -2000bp of the POP2 promoter, an HA-tag, and the complete 3'UTR to the poly-A tail for genetic reconstitution in murine macrophages. An HA-tag sequence was added to ensure a detectable protein epitope. **(B)** Induction of POP2 mRNA in two stable POP2 Tg J774A.1 cell clones (left panel, RT-PCR) and three stable POP2 Tg RAW264.7 clones (qPCR) after LPS (100 ng ml^{-1}) stimulation for 60 min (right panel, mean ± SD), representative of two similar experiments. **(C)** Level of TNF or mature IL-1β in pcDNA3 or POP2 Tg J774A.1 clone 4 following LPS (100 ng ml^{-1}) treatment for 24 h or plus ATP (5 mM) or nigericin (10μM) treatment for 30 min. Means ± SD are shown for two or more independent experiments. To determine statistical significance (panel C), Student's t-test was used. *$p<0.05$

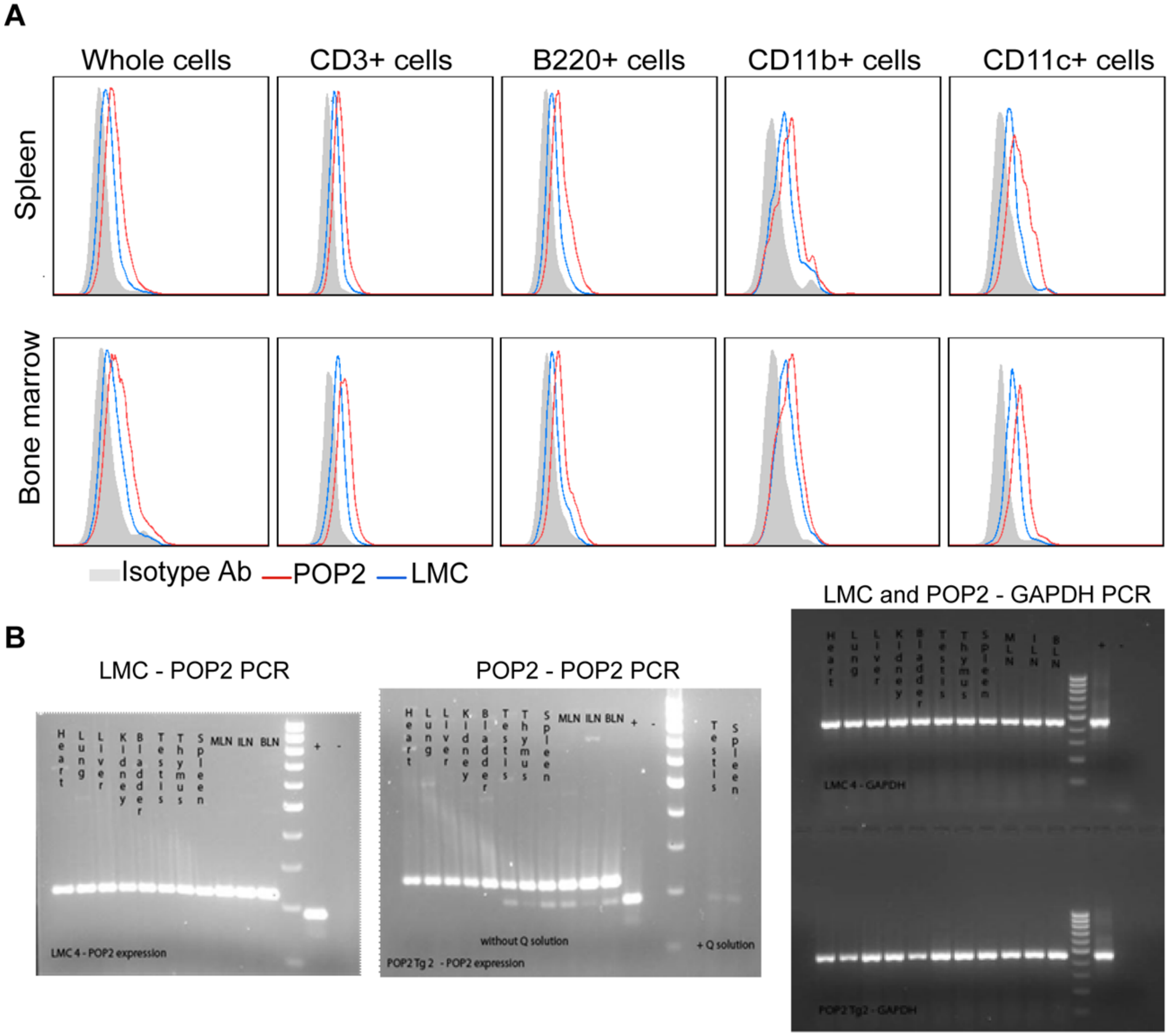

Supplementary Figure 3. **Flow cytometry analysis of POP2 expression in different subsets of immune cells, and full gels from Figure 1A. (A)** Single cell suspensions obtained from spleen or bone marrow aspirates of naïve POP2 and LMC mice were stained with lymphoid cell (CD3, CD4, and CD8) and myeloid cell markers (CD11b, CD11c, F4/80, and Gr-1) for 30 min. The cells were fixed in 1% paraformaldehyde, permeabilized and stained with POP2 polyclonal IgG antibody (sc-243843, Santa Cruz) or IgG control antibody for 30 min. Multi-parameter flow cytometry was performed on an LSRII (Becton Dickinson) and data were analyzed using FlowJo software (v10.0.1). Histograms are representative of two independent experiments. **(B)** Full gel photos referenced in Figure 1A.

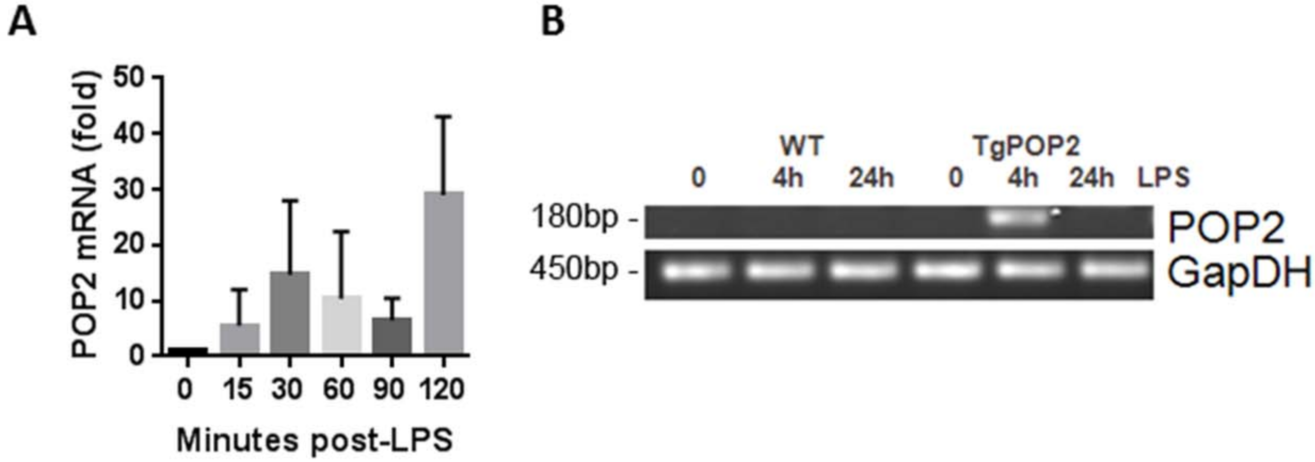

Supplementary Figure 4. **qPCR analysis of POP2 expression in murine primary macrophages treated with LPS.** (**A**) Macrophages (BMDM) were cultured in 6-well plates (1 x10^6 cells/well) and stimulated with LPS (100 ng ml^{-1}) for the times indicated. RNA was isolated using RNeasy purification columns (Qiagen) and treated with DNAase I. Quantitative PCR was performed using the SuperScript III Platinum SYBR Green One-Step qRT-PCR kit (Invitrogen). All reactions were run in triplicate. C_t values were normalized to β-actin as an internal control and relative copy numbers calculated by the standard 2^{-dCT} method. Data are from two independent experiments (mean ± SD). (**B**) Representative gel images showing POP2 expression: Semi-quantitative RT-PCR was performed using ~500ng of RNA and OneStep RT-PCR Kit (Qiagen) to detect POP2 expression in LPS stimulated BMDMs. Gel image is representative of two independent experiments.

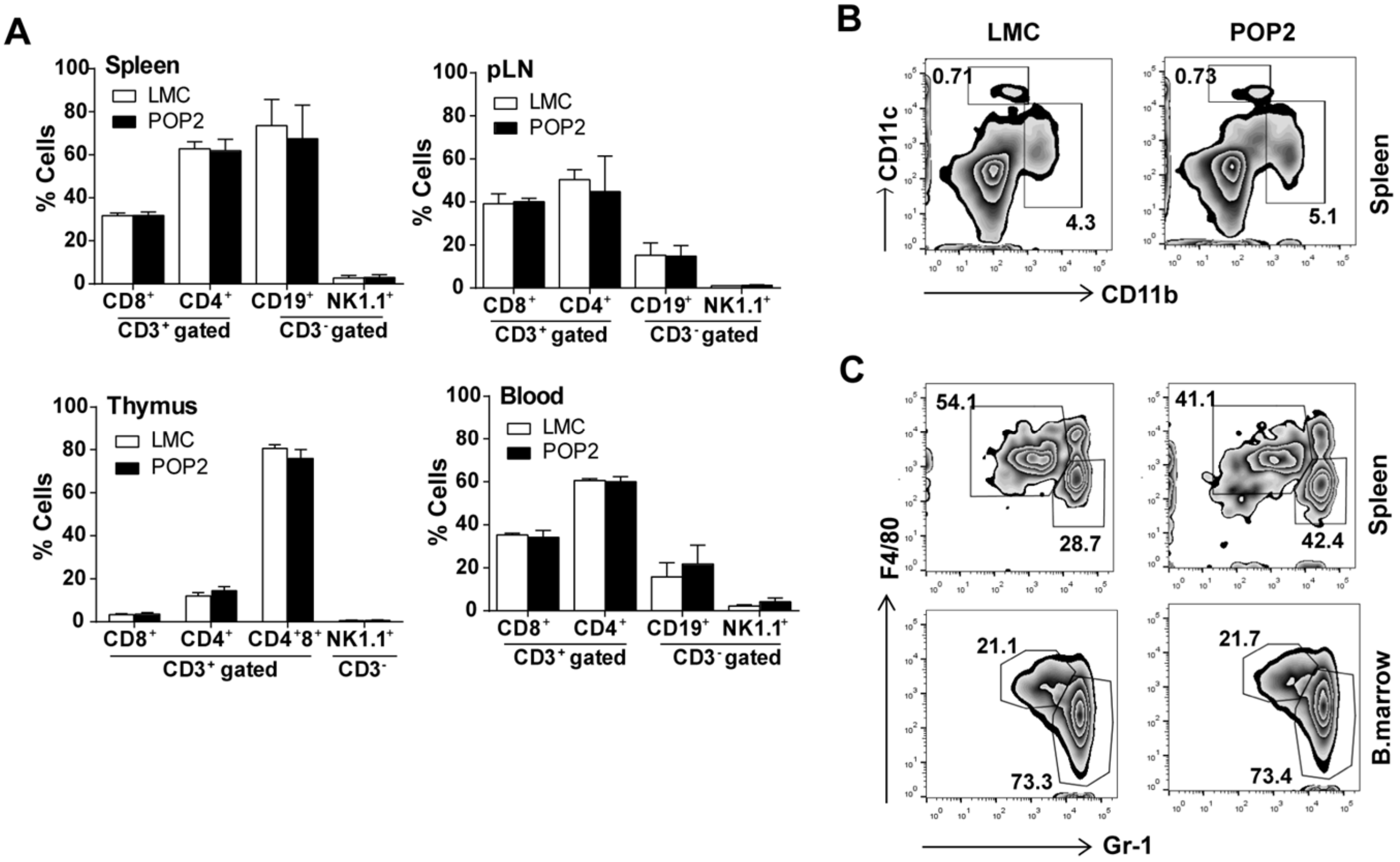

Supplementary Figure 5. **Immunophenotyping of immune cell subsets in POP2 and LMC mice.** (**A**) The frequencies of lymphoid cells in spleen, peripheral lymph nodes (pLN), thymus and blood. Data represent mean ± SD from two independent experiments using cells from POP2 (n=4) and LMC (n=4) mice. Student's t-test was used to determine statistical significance. No statistically significant difference was observed. (**B-C**) Representative flow plots for gating/analysis of myeloid cells: single cell suspensions obtained from spleen or bone marrow were stained with lymphoid cell (CD3, CD4, and CD8) and myeloid cell markers (CD11b, CD11c, F4/80, and Gr-1) for 30 min. The cells were fixed in 1% paraformaldehyde and analyzed in LSRII (Becton Dickinson). For Figure 1D, live cells were gated and analyzed for CD11b and CD11c expression to identify myeloid cells **(B)** and C11b+ cells were further gated for analysis of F4/80+ macrophages and Gr-1+ neutrophils **(C)**.

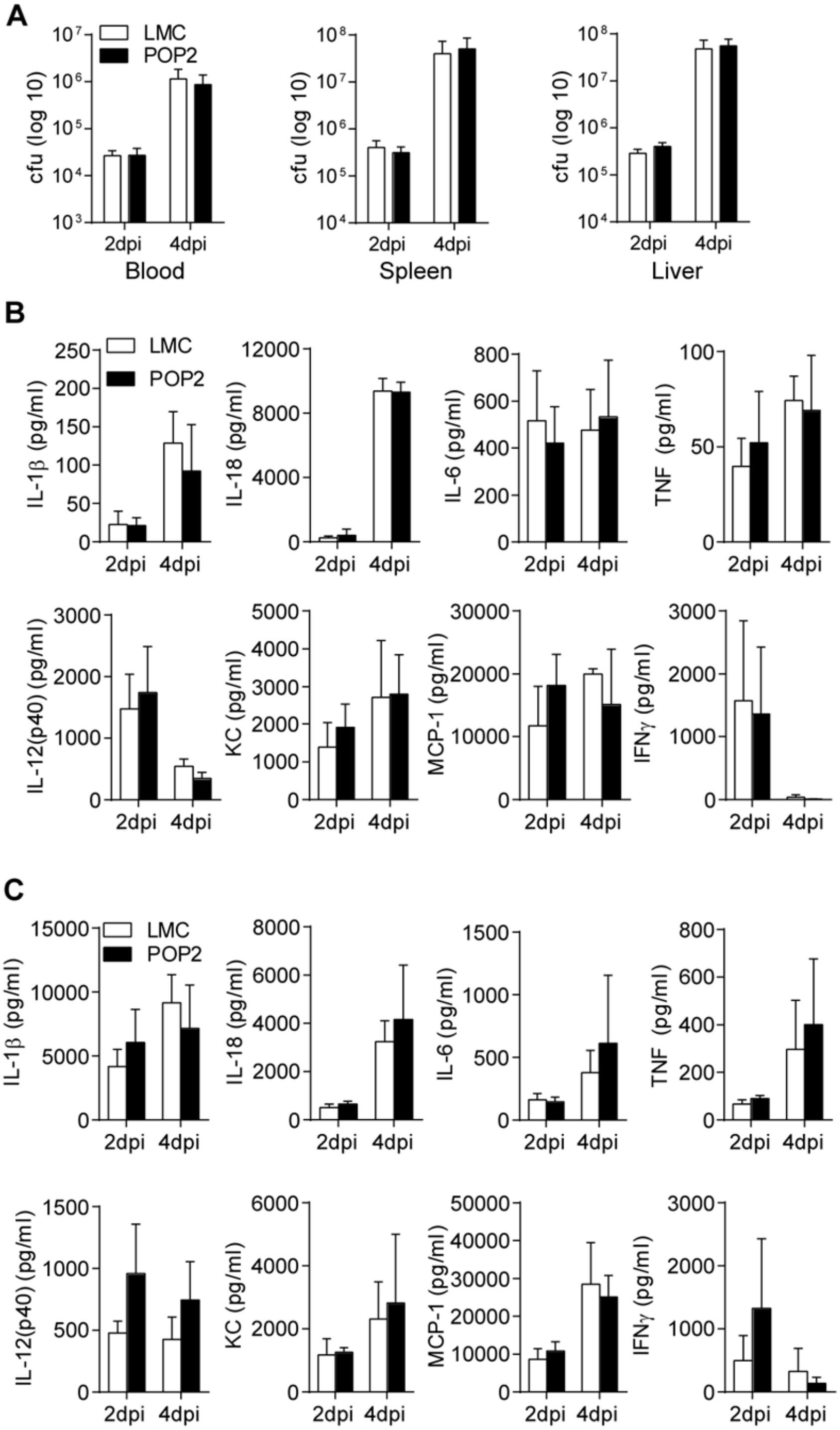

Supplementary Figure 6. **Bacterial burden and cytokine response to orogastric Salmonella infection of POP2 mice. (A)** Bacterial burden in blood, spleen, and liver of POP2 (n=4) and LMC (n=4) mice infected with Salmonella Typhimurium (10^6 cfu, orogastric route). **(B)** Levels of inflammatory cytokines measured in serum and **(C)** spleen homogenates. **(A-C)**. Means ± SD are shown and are representative of two independent experiments; Student's *t*-test was applied to analyze statistical differences between LMC and POP2 mice at the indicated time points, no statistically significant differences were detected.

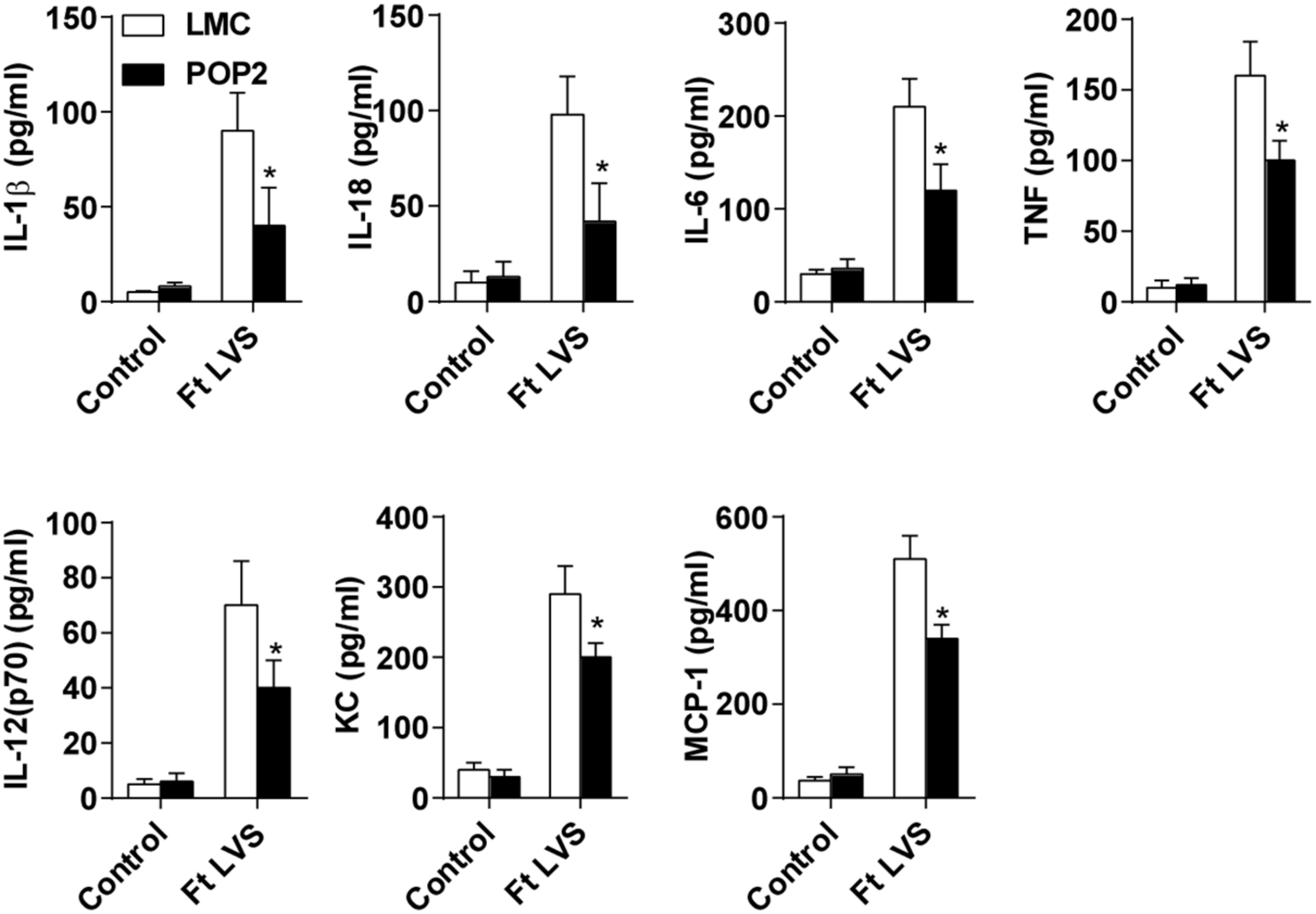

Supplementary Figure 7. **Serum cytokine levels in POP2 and LMC mice infected with *F. tularensis*:** Levels of the indicated cytokines in sera from *F. tularensis* LVS-infected (1000 cfu) POP2 (n=4) and LMC (n=4) mice were measured using a Luminex assay. Means ± SD are shown and are representative of two independent experiments. Statistical significance was evaluated using Student's t-test. *P<0.05

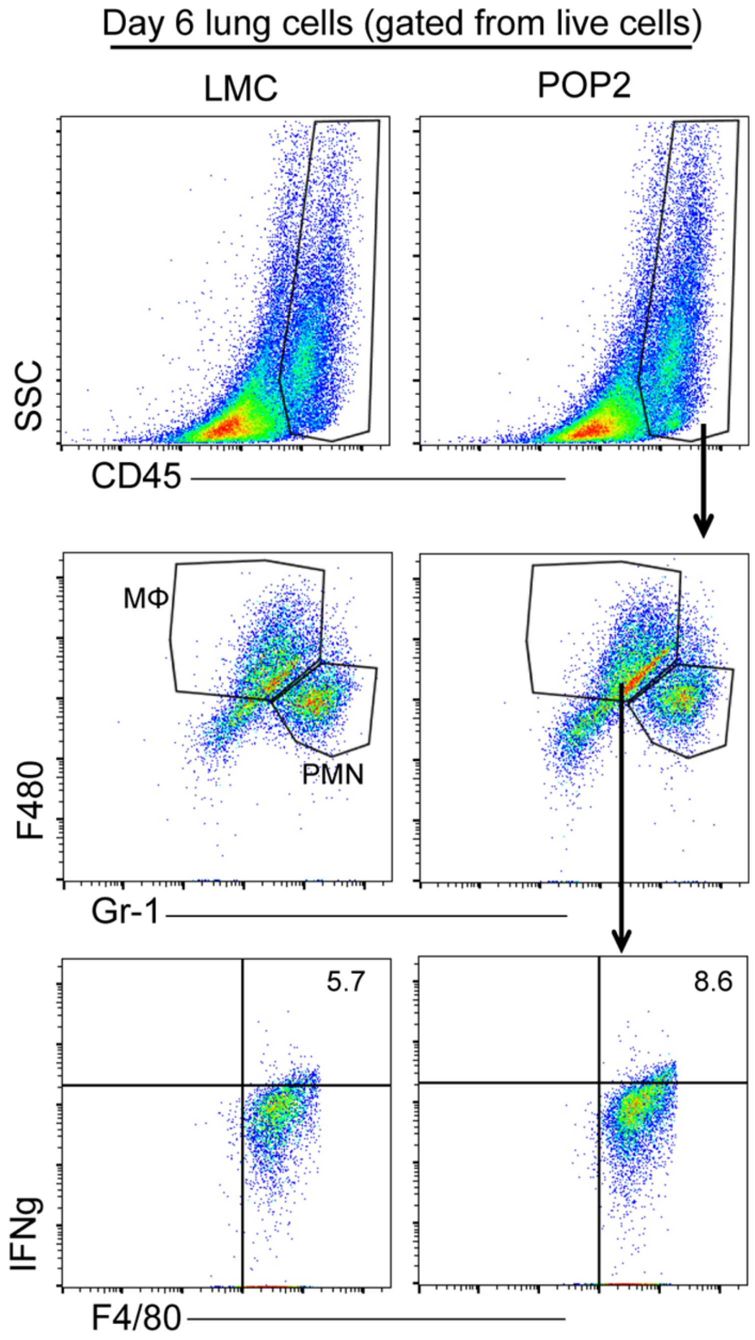

Supplementary Figure 8. Gating strategy for IFNγ expressing macrophages in Figure 6G. Representative flow plots show pattern for gating/analysis of myeloid cells and intracellular cytokine staining. Single cell suspensions obtained from spleen or lungs of Ft LVS-infected mice were stained with CD45, lymphoid cell (CD3, CD4, and CD8) and myeloid cell markers (CD11b, CD11c, F4/80, and Gr-1) for 30 min. The cells were fixed in 1% paraformaldehyde and were permeabilized and stained with specific cytokine (IFNγ) antibody or isotype control antibody for 30 min. Multi-parameter flow cytometry was performed on an LSRII (Becton Dickinson) and data were analyzed using FlowJo software (v10.0.1). First, live cells were gated and analyzed for CD45 to exclude non-haematopoietic cells. From CD45+ haematopoietic cells, myeloid cells were analyzed for intracellular expression of IFNγ in F4/80+ macrophages. Quadrants were established based on control antibody staining.

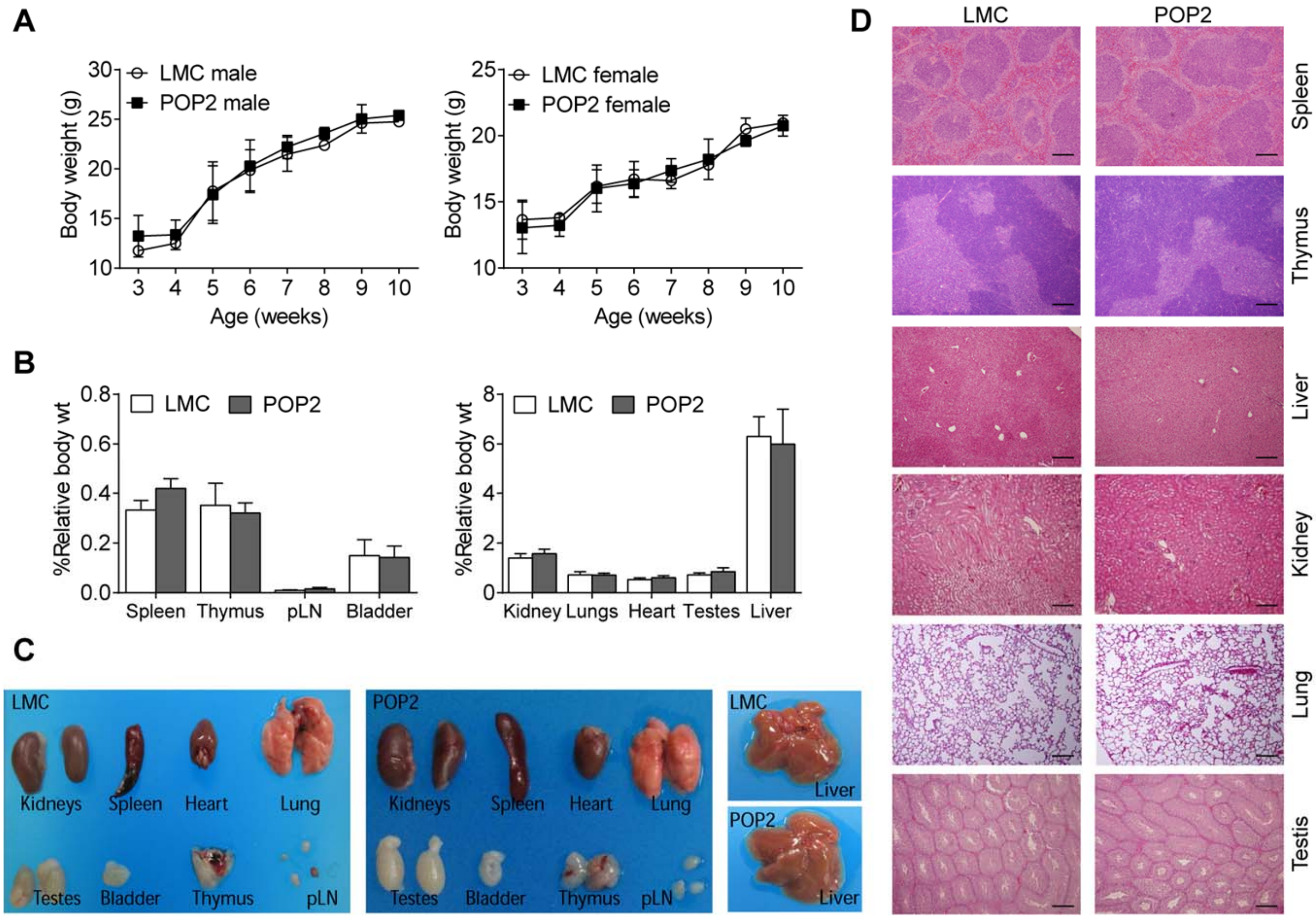

Supplementary Figure 9. (**A**) General phenotyping of POP2Tg mice: Body weights of male and female POP2 (n≥3) mice compared to LMC (n≥3) mice. Data represent mean ± SD for each age, genotype, and gender. **(B)** Weights of organs from POP2 and LMC mice shown as a percentage relative to total body weight. Data represent mean ± SD from POP2 (n=5) mice and LMC (n=6) mice, with significance tested using Student's t-test. No statistically significant differences were observed. (**C**) Gross evaluation of tissues of POP2 and LMC mice: representative images for gross anatomy of different tissues of POP2 and LMC mice showing normal structures. (**D**) Histological evaluation of tissues from POP2 and LMC mice: microscopic anatomy of tissue sections showing normal structures of spleen, thymus, liver, kidney, lung and testes. Tissue sections were stained with haematoxylin and eosin and visualized with light microscopy (scale bar = 20μm). Gross and microscopic images are representative of two independent experiments.

Organ and changes	Score
Lungs	
1) No lesions	0
2) Location of inflammation	
a) Peribronchiolar or perivascular	1
b) Alveolar lumen and alveolar wall	2
c) a + b	3
d) a+ b and exudates in bronchi/bronchiolar lumen	4
3) Type of cellular infiltrates in the inflammatory foci	
a) Neutrophils (a few)	1
b) Neutrophils (predominant) and monocytes/macrophages	2
b) Neutrophils and macrophages and lymphocytes (mixed infiltrates)	3
c) Mixed infiltrates and granulomatous foci,	4
4) Extent of inflammation in the lung parenchyma	
a) Patchy (small) inflammatory foci, few	1
b) Patchy inflammatory foci, many	2
c) Large inflammatory foci, many	3
d) Necrotizing inflammatory foci	4
5) Extent of necrotic changes	
a) Small necrotic foci, few	2
b) Small necrotic foci, many	3
c) Large necrotic foci	4
Spleen	
a) No lesions	0
a) Splenomegaly	2
b) Marginal zone thickening and red pulp inflammation	3
c) Granulomatous inflammation	4
Liver	
a) No lesions	0
b) Hepatic lobular infiltration by neutrophils and mononuclear cells	2
b) Granulomatous inflammation	3
c) Necrotizing and granulomatous inflammation	4

Supplementary Figure 10. Histopathology scoring criteria for microscopic lesions observed in Ft-infected tissues**.**
